# Supplementary material for: Enhanced BRAF engagement by NRAS mutants capable of promoting melanoma initiation
Source: Nat Commun. 2022 Jun 7;13:3153. doi: 10.1038/s41467-022-30881-9 (PMC9174180; doi:10.1038/s41467-022-30881-9)

Supplementary Information

Enhanced BRAF engagement by NRAS mutants capable of promoting melanoma initiation

Murphy *et al.*

Supplementary Material **Page**

Figure 1…………………………………………….2-4
Figure 2………………………………………………5
Figure 3………………………………………………6
Figure 4………………………………………………7
Figure 5………………………………………………8
Figure 6………………………………………………9
Figure 7……………………………………..……10
Figure 8……………………………………………..11
Figure 9……………………………………………..12
Figure 10……………………………………………13
Figure 11……………………………………………14
Figure 12……………………………………..…15-16

Table 1a…………………………………………….17
Table 1b…………………………………………….18
Table 1c…………………………………………….19
Table 1d…………………………………………….20
Table 1e…………………………………………….21
Table 1f…………………………………………….22
Table 2 ….………………………………………….23
Table 3a…………………………………………….24
Table 3b…………………………………………….25
Table 4a…………………………………………….26
Table 4b…………………………………………….27
Table 4c…………………………………………….28
Table 4d…………………………………………….29
Table 4e…………………………………………….30
Table 5a ……………………………………………31
Table 5b ……………………………………………32
Table 5c ……………………………………………3C

Supplementary Figures

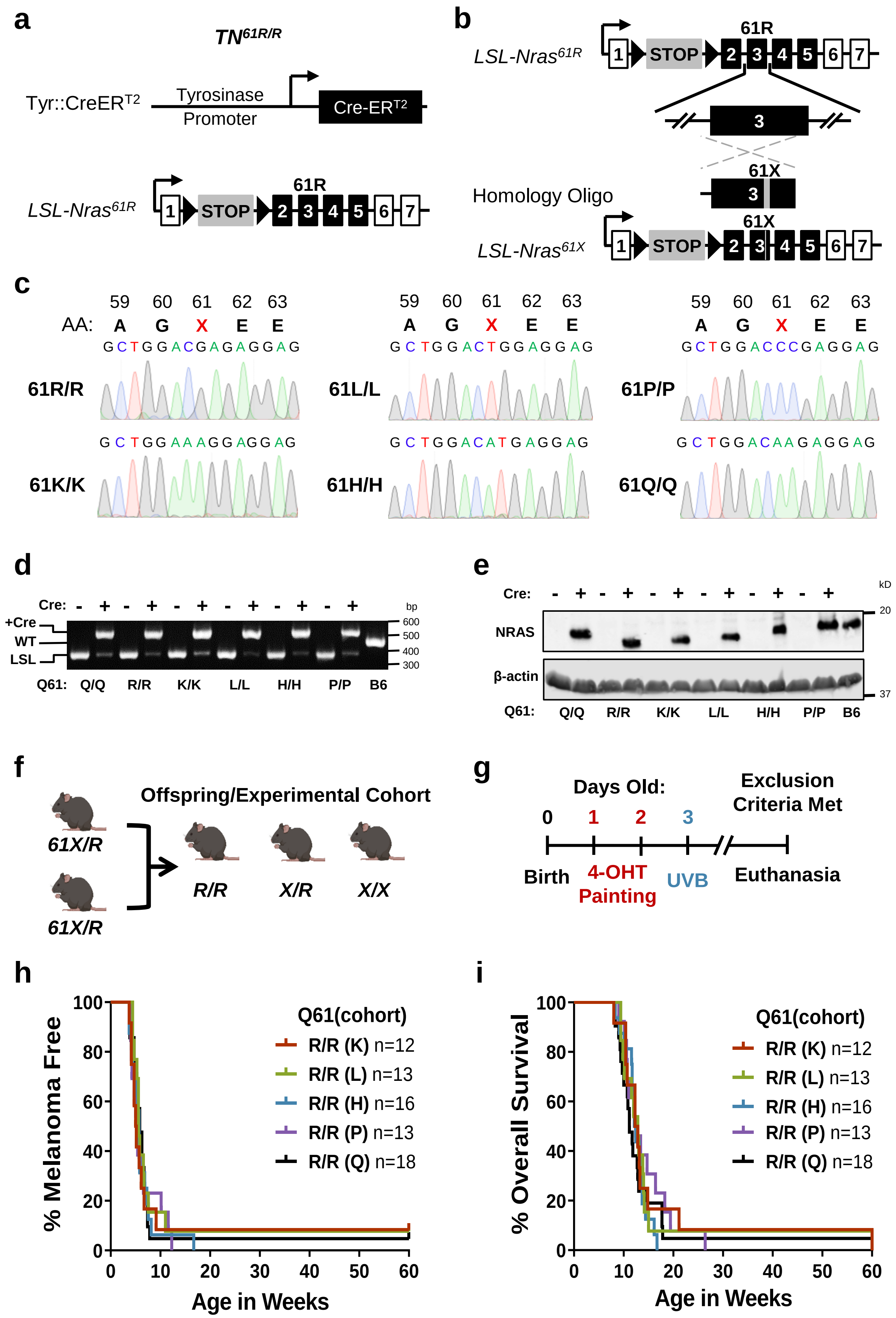

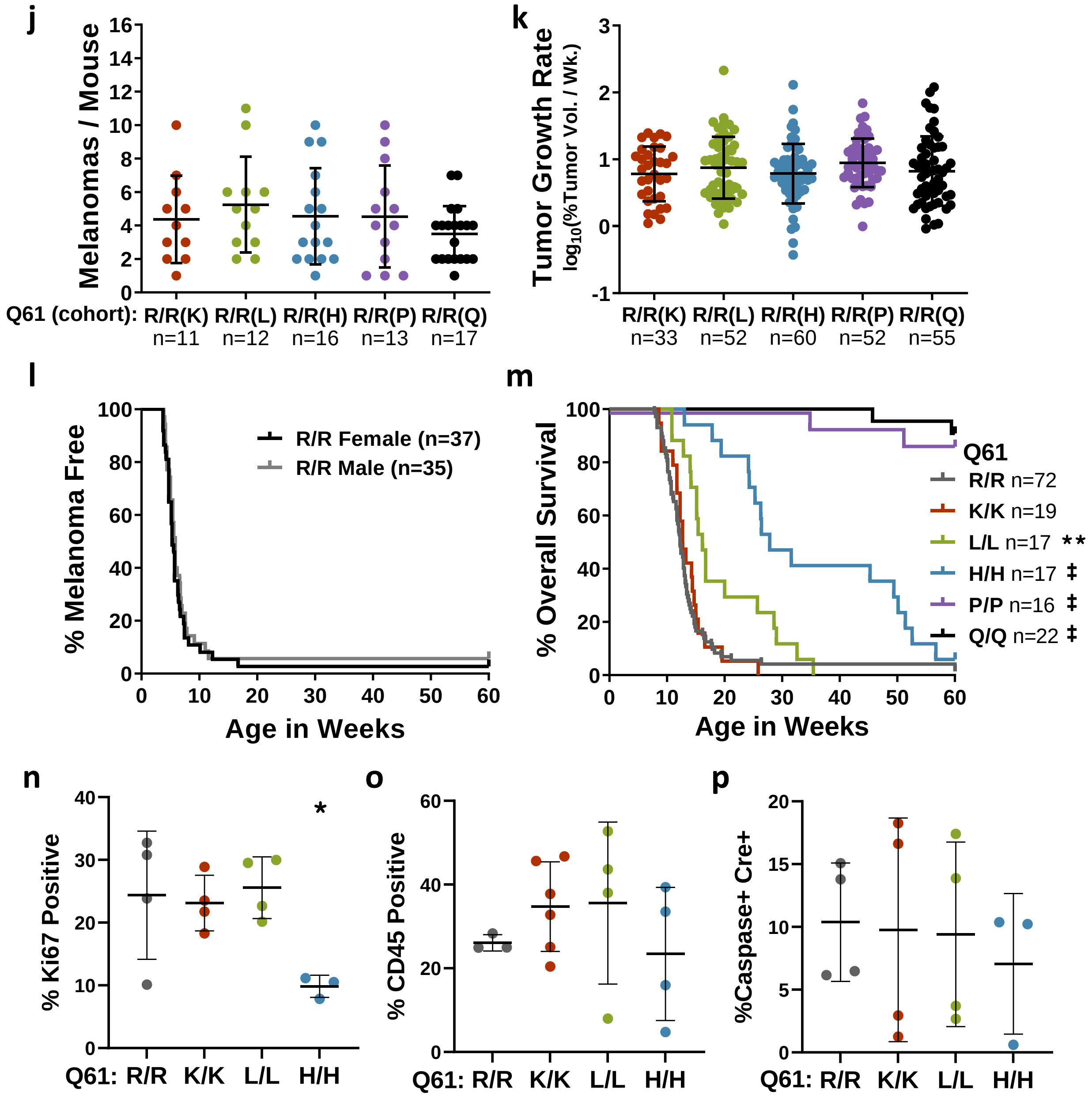
j
Melanomas / Mouse
Q61 (cohort): R/R(K) R/R(L) R/R(H) R/R(P) R/R(Q)
n=11 n=12 n=16 n=13 n=17
k
Tumor Growth Rate
log10(%Tumor Vol. / Wk.)
R/R(K) R/R(L) R/R(H) R/R(P) R/R(Q)
n=33 n=52 n=60 n=52 n=55
l
% Melanoma Free
R/R Female (n=37)
R/R Male (n=35)
Age in Weeks
m
% Overall Survival
Q61
R/R n=72
K/K n=19
L/L n=17 **
H/H n=17 ‡
P/P n=16 ‡
Q/Q n=22 ‡
Age in Weeks
n
% Ki67 Positive
*
Q61: R/R K/K L/L H/H
o
% CD45 Positive
Q61: R/R K/K L/L H/H
p
%Caspase+ Cre+
Q61: R/R K/K L/L H/H

Supplementary Figure 1: Mouse model generation and experimental design for evaluating the tumorigenicity of NRAS codon 61 mutants. **a**, Diagram of alleles present in the $TN^{61R/R}$ model. $TN^{61R/R}$ mice are homozygous for a melanocyte-specific Cre-ER^{T2} transgene (Tyr::Cre-ER^{T2}) and the *LSL-Nras*^{61R} conditional knock-in allele. **b**, Schematic representation of the CRISPR-Cas9-directed strategy used to alter *Nras* codon 61 in $TN^{61R/R}$ embryos. **c**, Sequencing chromatograms of *Nras* exon 3 in MEF DNA isolated from each homozygous $TN^{61X/X}$ model. AA: amino acid. **d**, PCR screen for *LSL* recombination in genomic DNA from $TN^{61X/X}$ MEFs 72 hours post-infection with adenoviral eGFP 72 hours post-infection with adenoviral eGFP (-Cre, control) or Cre (+Cre). **e**, Immunoblot of protein lysates isolated from MEFs 5 days after adenoviral infection as described in 'D'. **f**, Breeding scheme used to generate experimental litters of $TN^{61X/X}$ mice. **g**, Diagram of the experimental treatment protocol. Topical application of 20 mM 4-hydroxytamoxifen (4-OHT) on postnatal days 1 and 2 was used to induce CreER^{T2} activity. Ultraviolet-B (UVB) irradiation was administered on postnatal day 3 as a single, 4.5 kJ/m^2 dose using a fixed position, 16W, 312 nm UVB light source. **h-k**, Melanoma-free survival (**h**), overall survival (**i**), total tumor burden (**j**) and tumor growth rates (**k**) for $TN^{61R/R}$ mice from each experimental cohort. Tumor burden and growth rate dot plots are presented as mean values +/- SD. The following number biologically independent animals were evaluated per genotype (R/R (K) = 12, R/R (L) = 13, R/R (H) = 16, R/R (P) = 13, R/R (Q) = 18). **l**, Melanoma-free survival of male and female $TN^{61R/R}$ mice. The following number biologically independent animals were evaluated per sex (male = 37, female = 35) No significant differences were detected between cohorts or sexes in log-rank (Mantel-Cox) (**h-i, l**) and one-way ANOVA analyses with Tukey's multiple comparison test (**j-k**). **m**, Overall survival of $TN^{61X/X}$ mice homozygous for the indicated *LSL-Nras* alleles. n ≥ 16 biologically independent animals were evaluated per genotype. The following number biologically independent animals were evaluated per genotype (61R = 72, 61K = 19, 61L = 17, 61H = 17, 61P = 16, 61Q = 22). Log-rank (Mantel-Cox) tests were used to compare the overall survival of each genotype. $TN^{X/X}$ samples statistically different from $TN^{61R/R}$ are indicated in the figure. **n-p**, Quantification of immunohistochemistry (IHC) staining for Ki67 (**n**), CD45 (**o**), or cleaved Caspase 3 (**p**) in *TN* melanoma tissue. The following number of biologically independent samples were evaluated per genotype (Ki67: R/R = 4, K/K =4 , L/L = 4, H/H = 3; CD45: R/R = 3, K/K = 6, L/L = 4, H/H = 4; Caspase: R/R = 4, K/K = 4, L/L = 4, H/H = 3). One-way ANOVA with a Dunnet's T3 multiple comparison test was used to compare staining intensities between each genotype and $TN^{61R/R}$. Each dot represents one biological replicate. Adjusted p-values for all comparisons can be found in Supplementary Table 1a&d. * $p< 0.05$, ** $p< 0.01$, † $p< 0.001$, ‡ $p< 0.0001$. Source data are provided as a Source Data file.

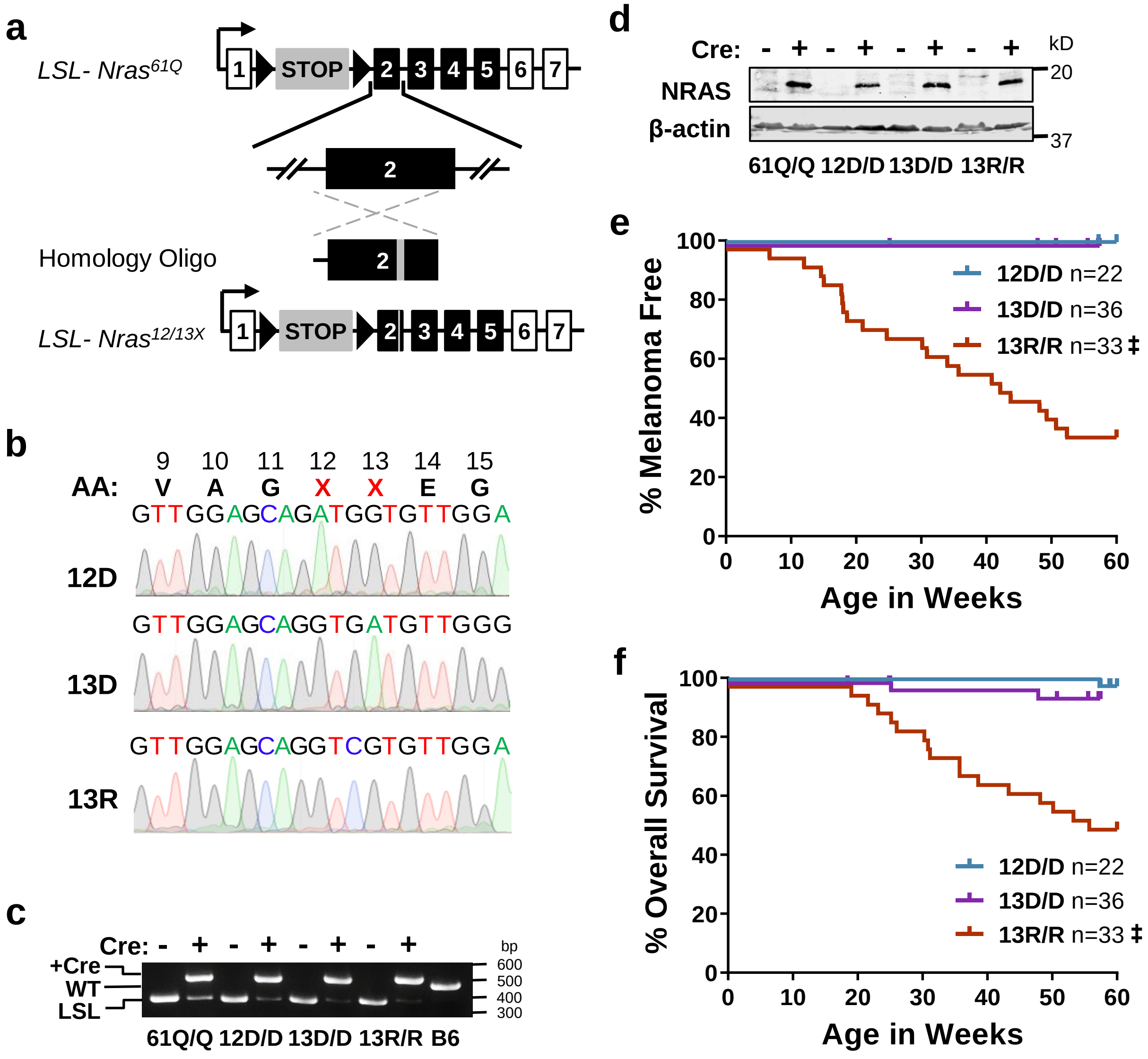

Supplementary Figure 2: Generation and evaluation of melanoma formation in NRAS codon 12 and 13-mutant mouse models. **a**, Schematic representation of the CRISPR-Cas9-directed mutagenesis strategy used to modify *Nras* codon 12 or 13 in $TN^{61Q/Q}$ embryos. **b**, Sequencing chromatograms of exon 2 in tissue DNA obtained from each homozygous $TN^{12X/X}$ or $TN^{13X/X}$ model. A silent G/A mutation in codon 15 occurred during the generation of the *LSL-Nras*^{12D} and *LSL-Nras*^{13R} alleles. **c-d**, Homozygous $TN^{12X/X}$ or $TN^{13X/X}$ MEFs were infected with adenovirus as described in Figure S1D. PCR shows the recombination of each *LSL-Nras* allele (**c**) and an immunoblot confirms protein expression (**d**). **e-f**, Melanoma-free survival (**e**) and overall survival (**f**) of mice expressing the indicated melanocyte-specific NRAS codon 12 or 13 mutants. The following number biologically independent animals were evaluated per genotype (12D/D = 22, 13D/D = 36, 13R/R = 33). Log-rank (Mantel-Cox) tests were used to compare each genotype to $TN^{12D/D}$. Adjusted p-values for all comparisons can be found in Supplementary Table 1f. ‡ p< 0.0001. Source data are provided as a Source Data file.

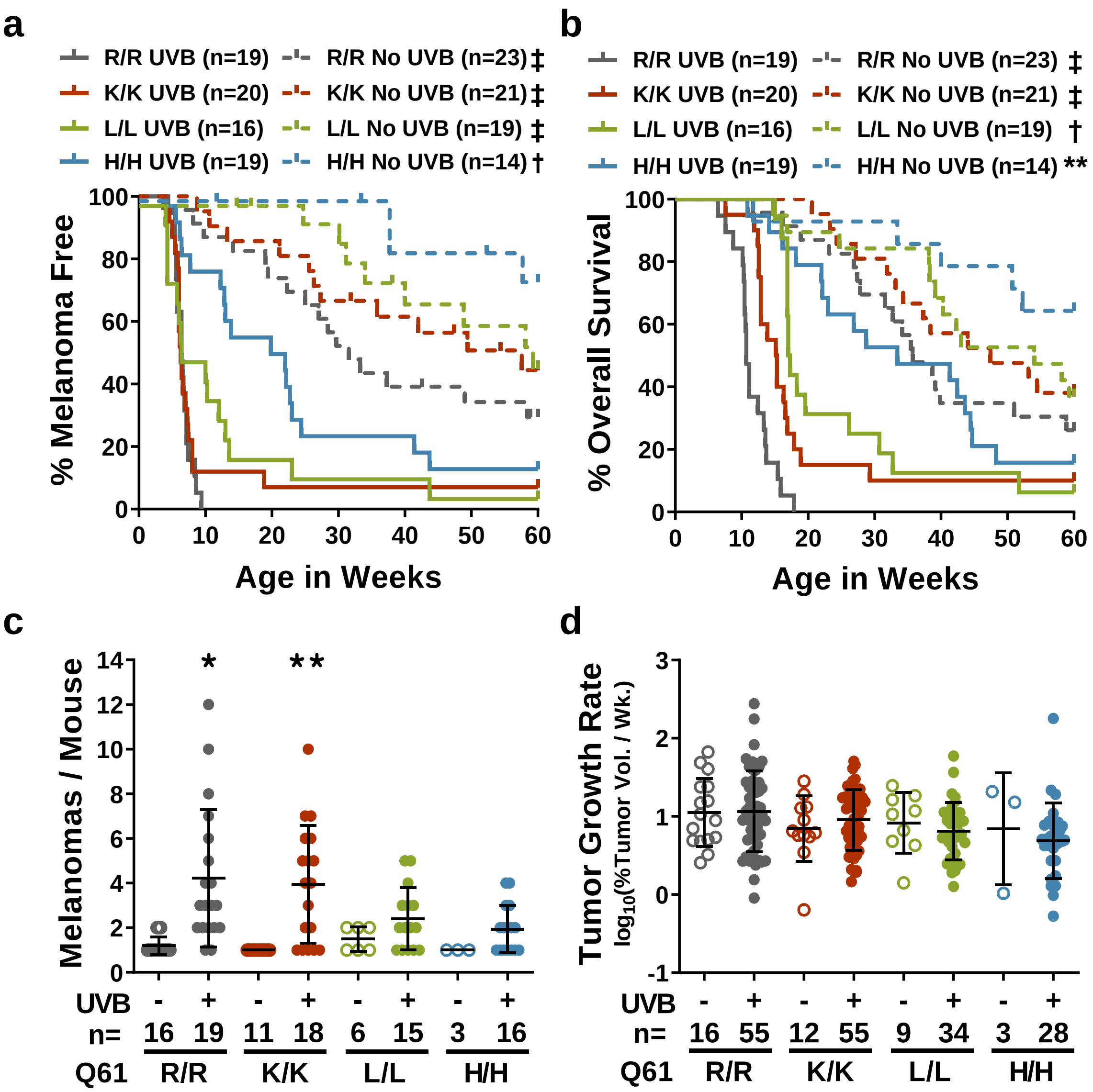

Supplementary Figure 3: NRAS mutants similarly cooperate with UVB irradiation to accelerate melanoma onset. a-d, Melanoma-free survival (**a**), overall survival (**b**), total tumor burden (**c**) and tumor growth rates (**d**) for TN$^{61X/X}$ mice treated with mock or UVB irradiation on postnatal day 3. Tumor burden and growth rate dot plots are presented as mean values +/- SD. The following number biologically independent animals were evaluated per genotype (UVB: R/R = 19, K/K = 20, L/L = 16, H/H = 19; No UVB: R/R = 23, K/K = 21, L/L = 19, H/H = 14). Log-rank (Mantel-Cox) (**a-b**) or one-way ANOVA (**c-d**) with a Dunnet T3 multiple comparisons test was used to compare measurements between UVB- and mock-treated mice within the same genotype. Adjusted p-values for all comparisons can be found in Supplementary Table 1e. * p< 0.05, ** p< 0.01, † p< 0.001, ‡ p< 0.0001. Source data are provided as a Source Data file.

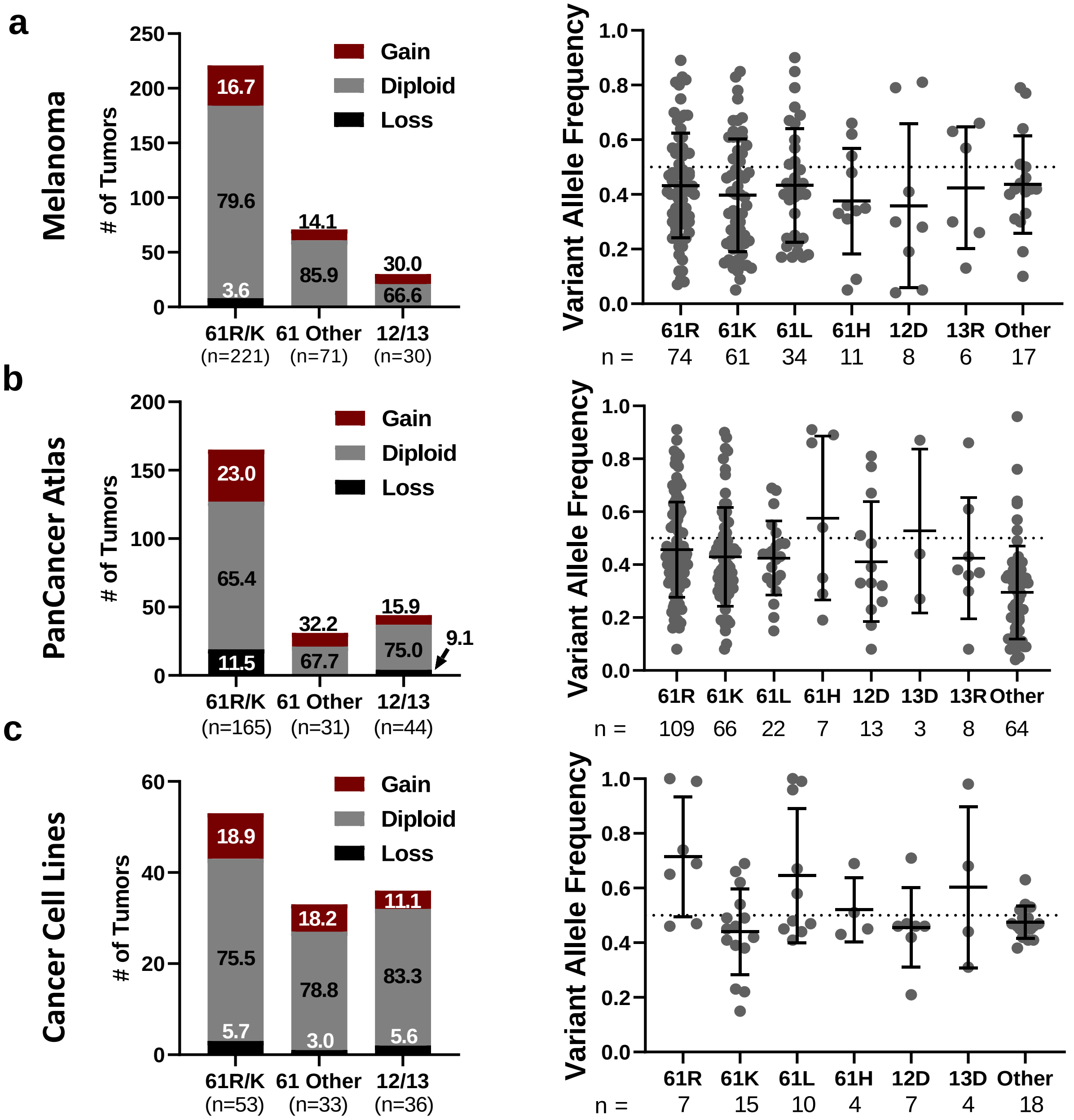

Supplementary Figure 4: Loss-of-heterozygosity is rare in NRAS-driven malignancies. **a-c**, NRAS copy number alterations (left) and variant allele frequencies (right) in NRAS-mutant melanomas from the MSKCC Melanoma dataset (**a**), NRAS-mutant malignancies from the TCGA PanCancer (**b**), and NRAS-mutant cell lines from the TCGA Cancer Cell Line dataset (**c**). Dot plot data are presented as mean values +/- SD where each dot represents one biological replicate. Source data are provided as a Source Data file.

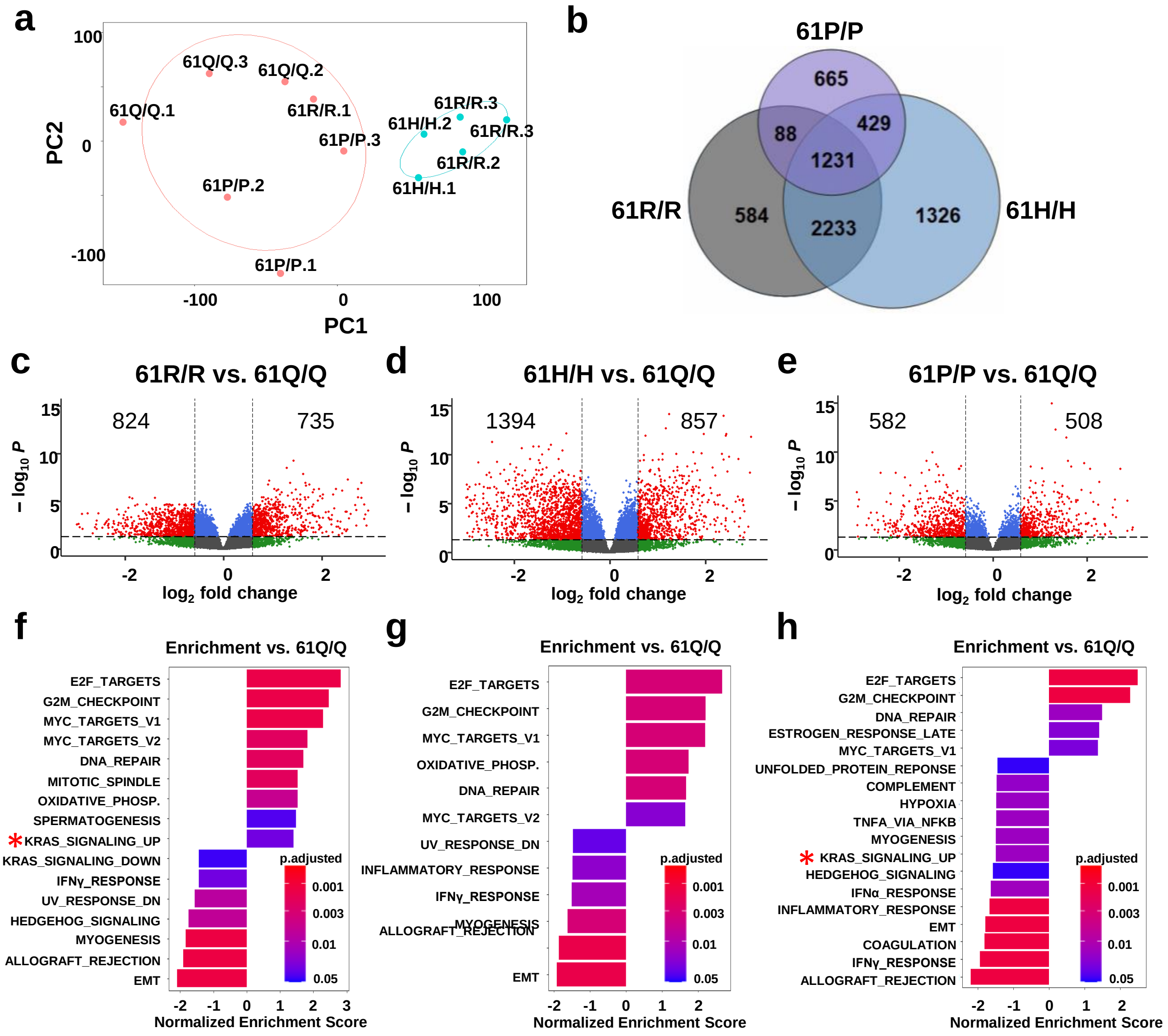

Supplementary Figure 5: Melanomagenic NRAS mutants drive the enrichment of hallmark genes associated with MYC, E2F and KRAS signaling. a, Principal component analysis of $TN^{61X/X}$ MEF samples analyzed by RNA-seq. Samples were grouped based on hierarchical clustering. Three biological replicates per genotype were used for analyzed. **b**, Venn diagram depicting the overlap of genes with differential expression in homozygous $NRAS^{61Q}$ MEFs and MEFs expressing each of the denoted $NRAS^{61X}$ mutants. Three biological replicates were used for each genotype. **c-e**, Volcano plots of differentially expressed genes in $TN^{61R/R}$ (**c**), $TN^{61H/H}$ (**d**) or $TN^{61P/P}$ (**e**) MEFs versus $TN^{61Q/Q}$ MEFs. **f-h**, Bar plots showing the enrichment of Hallmark gene sets (p-adjusted < 0.05) in $TN^{61R/R}$ (**f**), $TN^{61H/H}$ (**g**) or $TN^{61P/P}$ (**h**) MEFs versus $TN^{61Q/Q}$ MEFs. Three biological replicates were used for each genotype.

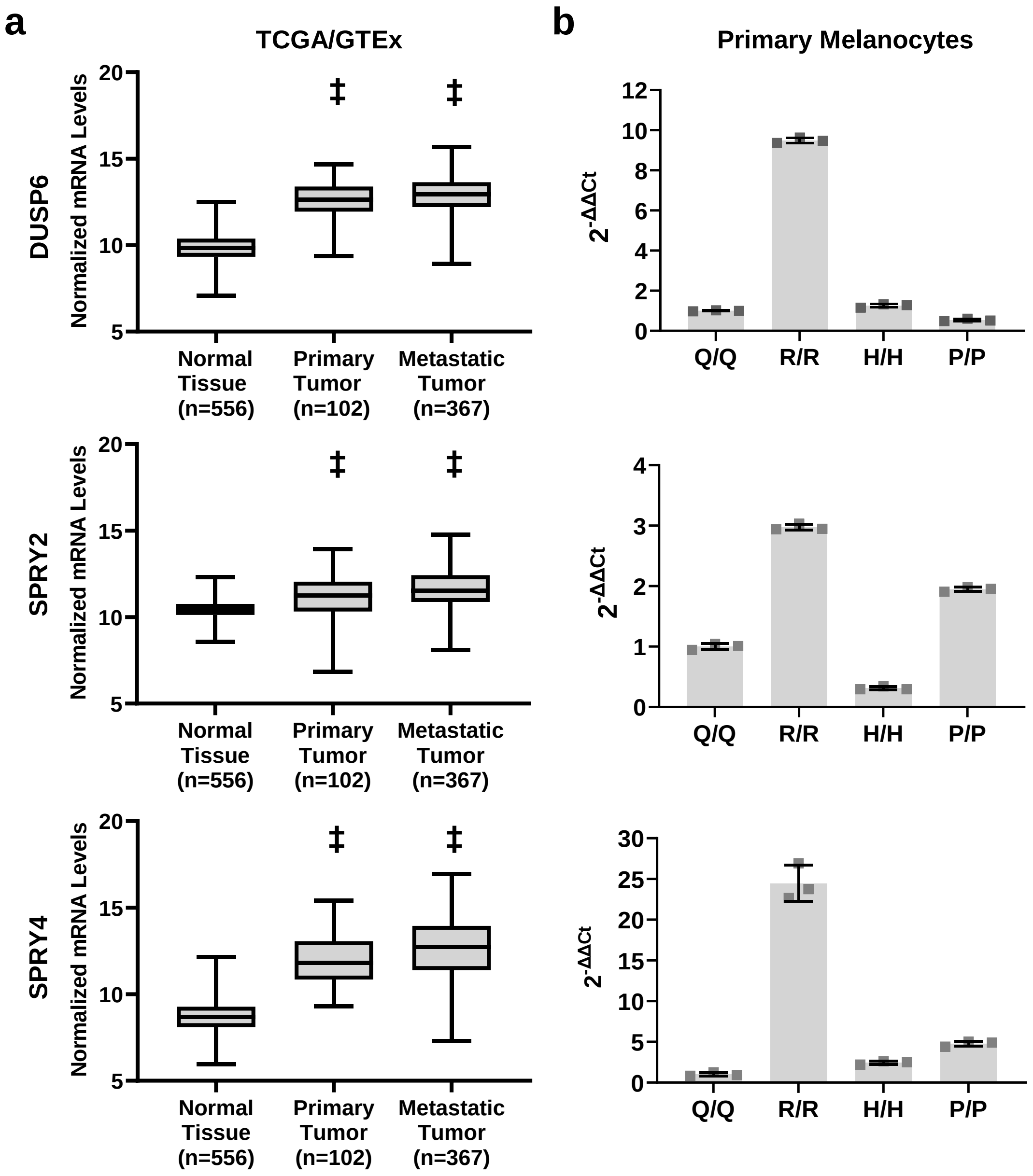

Supplementary Figure 6: Feedback inhibitors of the MAPK pathway are enriched in human melanoma and primary melanocytes expressing melanomagenic NRAS^{61R}. a, Box plots of *DUSP6* (top), *SPRY2* (middle), and *SPRY4* (bottom) expression in normal skin tissue (GTEX) versus primary and metastatic cutaneous melanoma (TCGA). Data were obtained from the UCSC Xena platform. Boxplot center line – mean, Bounds of Boxplot– Quartile 1 and 3 of data, Boxplot whiskers – min and max values. One-way ANOVA with a Dunnet's multiple comparison test was used to compare mRNA levels between tumor samples and normal tissues. ‡ $p< 0.0001$. **b**, Bar plots of qPCR data measuring *Dusp6* (top), *Spry2* (middle), or *Spry4* (bottom) mRNA expression in *TN* mouse melanocytes expressing the denoted NRAS mutants. n = 3 technical replicates ran over 1 independent experiment. Data are presented as mean values +/- SD. Source data are provided as a Source Data file.

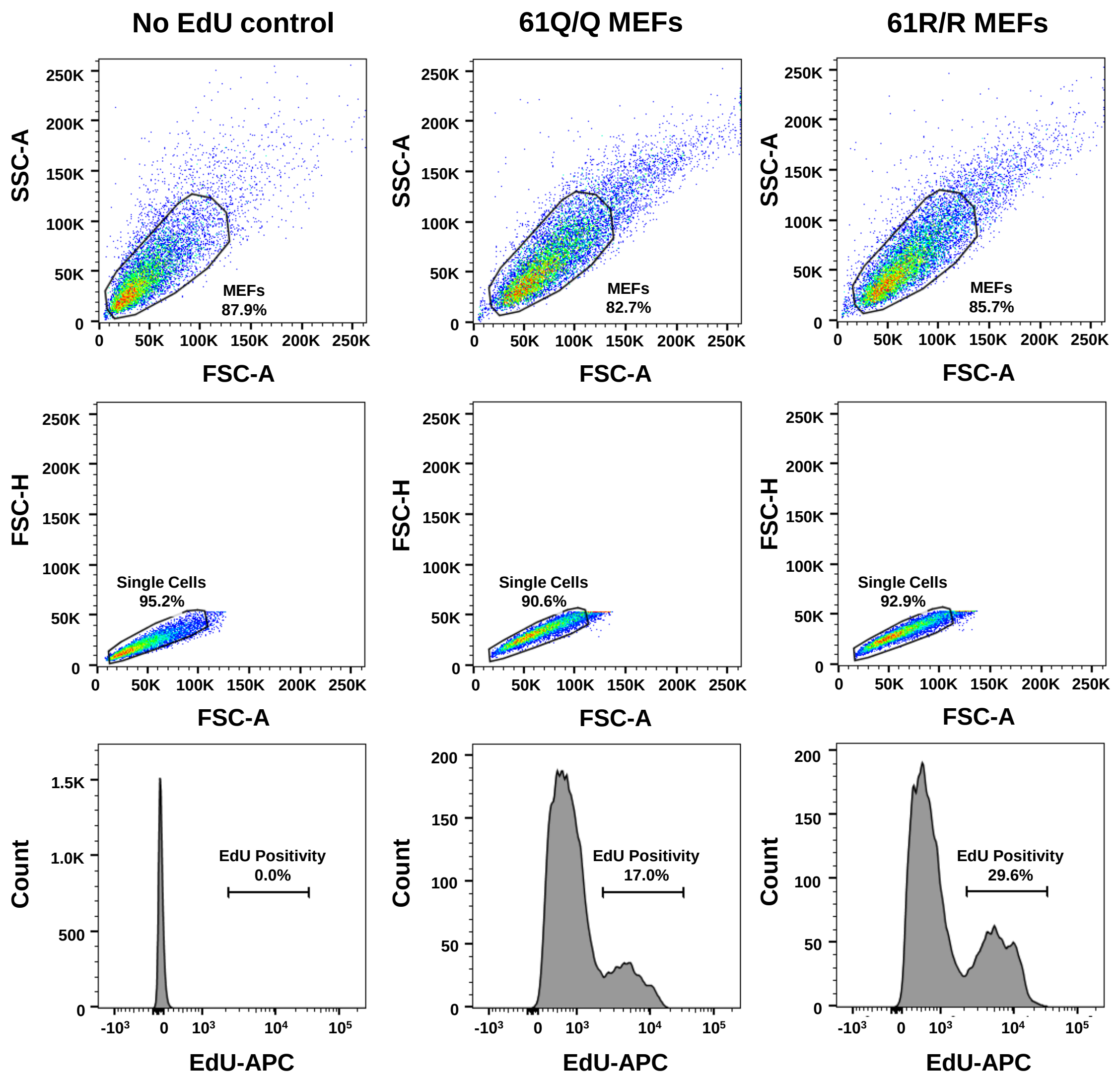

Supplementary Figure 7: Gating strategy for flow cytometric analysis of *in vitro* EdU incorporation in MEFs. MEFs, labeled with EdU conjugated to Alexa Fluor 555, were analyzed on a BD LSR Fortessa flow cytometer. The initial population of MEFs was selected by gating based on FSC-A and SSC-A (top). Cell doublets were removed by gating for single cells in a FSC-H by FSC-A plot (middle). Finally, a histogram of counts by APC-A intensity was used to determine the percent of EdU positive cells in each population (bottom).

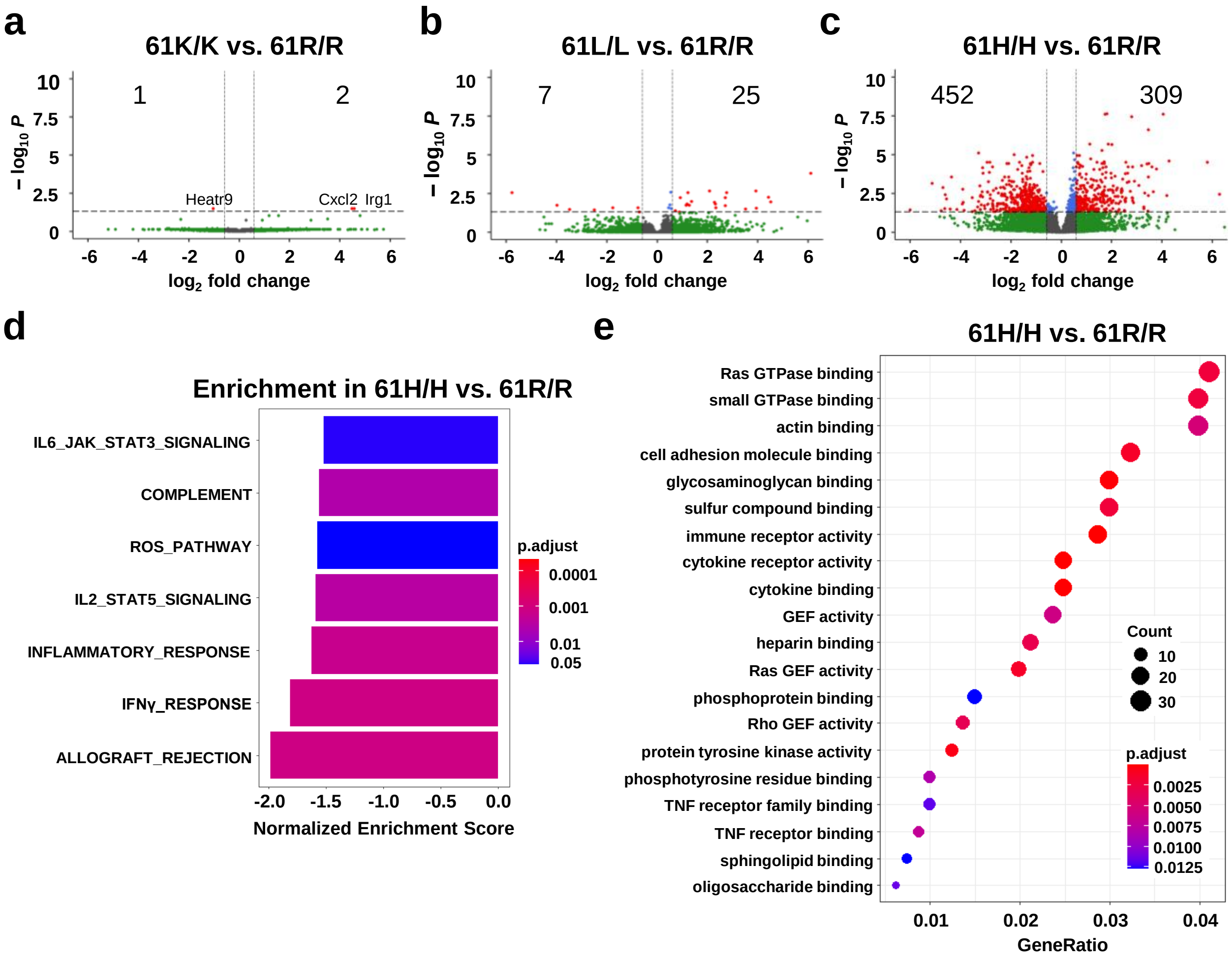

Supplementary Figure 8: Transcriptomic alterations between strong and weak drivers of melanoma center around RAS GTPase binding and immune regulation. a-c, Volcano plot depicting differentially expressed genes in $TN^{61R/R}$ versus $TN^{61K/K}$ (**a**), $TN^{61L/L}$ versus $TN^{61R/R}$ (**b**), or $TN^{61H/H}$ versus $TN^{61R/R}$ (**c**) melanomas. Data for each genotype was taken from 3 biological replicates. **d**, Bar plot illustrating the differential enrichment of Hallmark gene sets (p-adjusted < 0.05) in $TN^{61H/H}$ versus $TN^{61R/R}$ melanomas generated using gene set enrichment analysis (GSEA). **e**, Dot plot of gene sets associated with molecular functions that are differentially regulated (p-adjusted < 0.05) between three $TN^{61H/H}$ and three $TN^{61R/R}$ melanomas by Gene Ontology (GO) analysis.

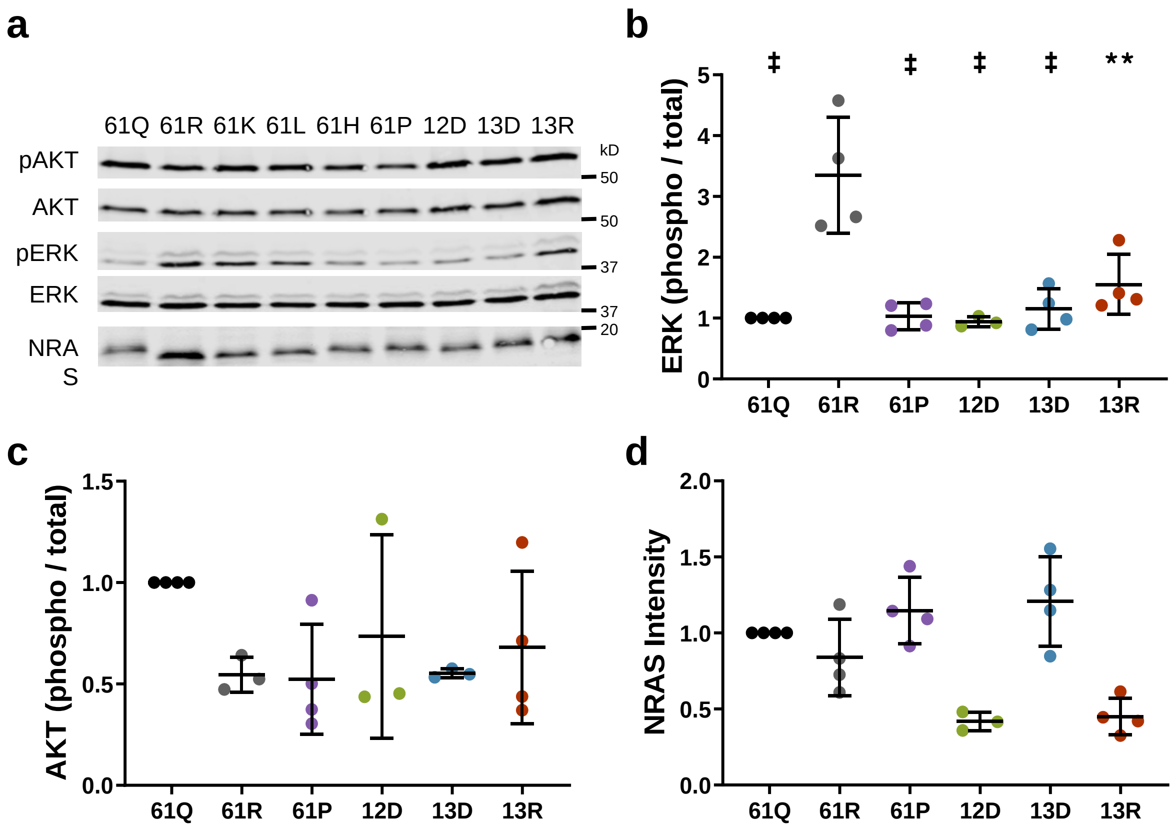

Supplementary Figure 9: Reduced MAPK>ERK activation by NRAS codon 12 and 13 versus NRAS codon 61 mutants. a, Representative immunoblot of protein lysates isolated from MEFs homozygous for the indicated NRAS mutants. **b-d**, Dot plots showing the quantification of ERK activation (**b**), AKT activation (**c**) or NRAS expression (**d**) in replicate immunoblots. Dot plot data are presented as mean values +/- SD where each dot represents one biological replicate. The following number of biologically independent replicates per genotype were examined over 4 independent experiments (61Q = 4, 61R = 4, 61P = 4, 12D = 3, 13D = 4, 13R = 4). One-way ANOVA with a Tukey's post-test was used to compare data between each genotype. NRAS mutant samples statistically different from NRAS$^{61R/R}$ samples are indicated in the figure. Adjusted p-values for all comparisons can be found in Supplementary Table 3b. ** p< 0.01, ‡ p< 0.0001. Source data are provided as a Source Data file.

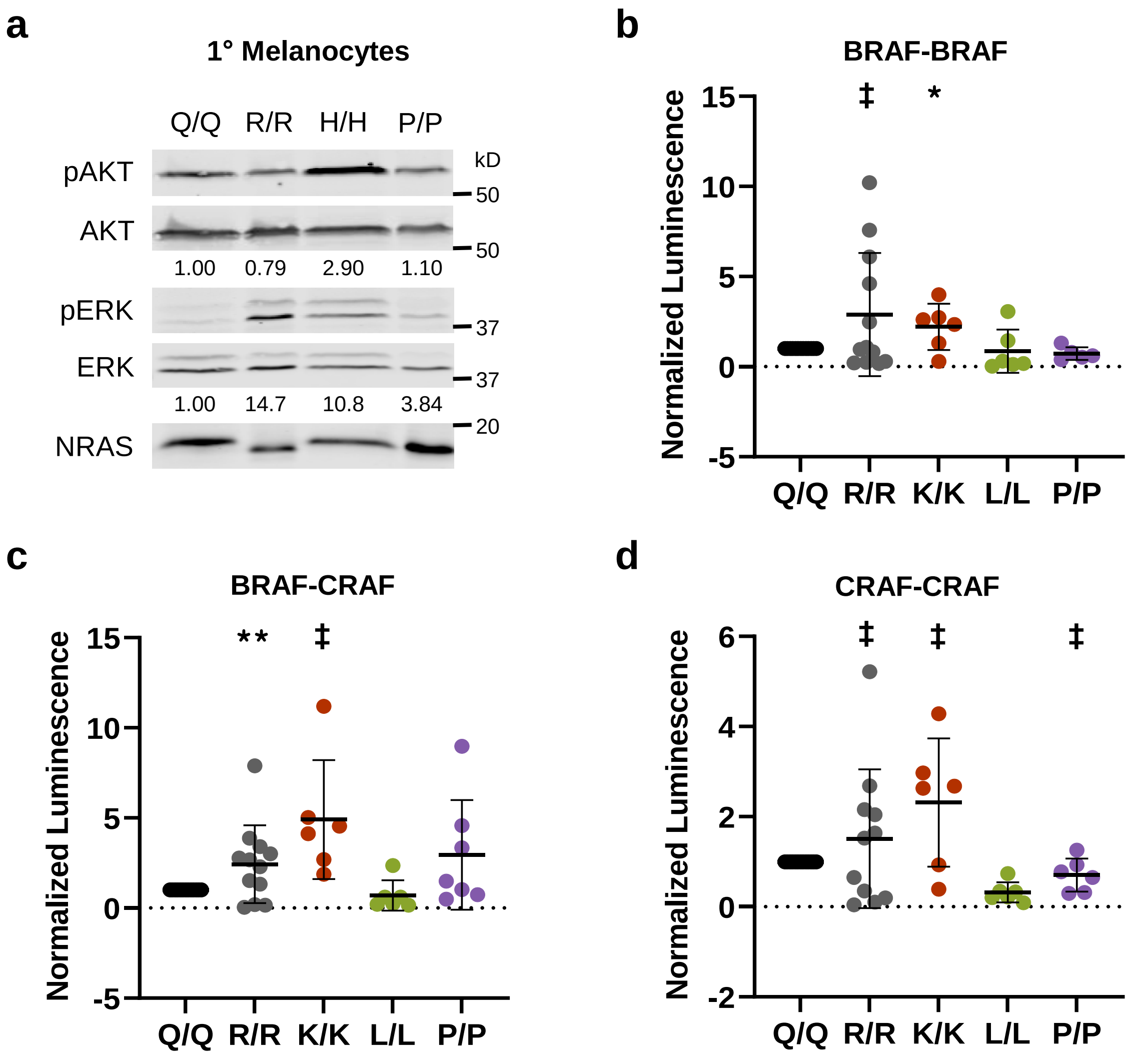

Supplementary Figure 10: Melanomagenic NRAS mutants drive enhanced MAPK signaling and RAF dimerization in primary melanocytes. a, Immunoblot of protein lysates isolated from primary melanocytes homozygous for the indicated NRAS mutants. Values represent quantification of AKT or ERK activation normalized to wildtype NRAS (Q/Q). **b-d**, Dot plot of normalized luminescence intensity in $TN^{61X/X}$ primary melanocytes infected with adenovirus expressing BRAF-LgBiT and BRAF-SmBiT (**b**), BRAF-LgBiT and Craf-SmBiT (**c**), or CRAF-LgBiT and CRAF-SmBiT (**d**). Luminescence intensity was normalized to crystal violet staining for each well. Dot plot data are presented as mean values +/- SD where each dot represents one biological replicate. The following number of biologically independent replicates per genotype were examined over 6 independent experiments (Q/Q = 12, R/R = 12, K/K = 6, P/P = 6, L/L = 6). One-way ANOVA with a Dunnet T3 multiple comparisons test was used to compare luminescence intensity from each codon 61 mutant to wildtype, $TN^{61Q/Q}$ melanocytes. Adjusted p-values for all comparisons can be found in Supplementary Table 4c. * $p< 0.05$, ** $p< 0.01$, ‡ $p< 0.0001$. Source data are provided as a Source Data file.

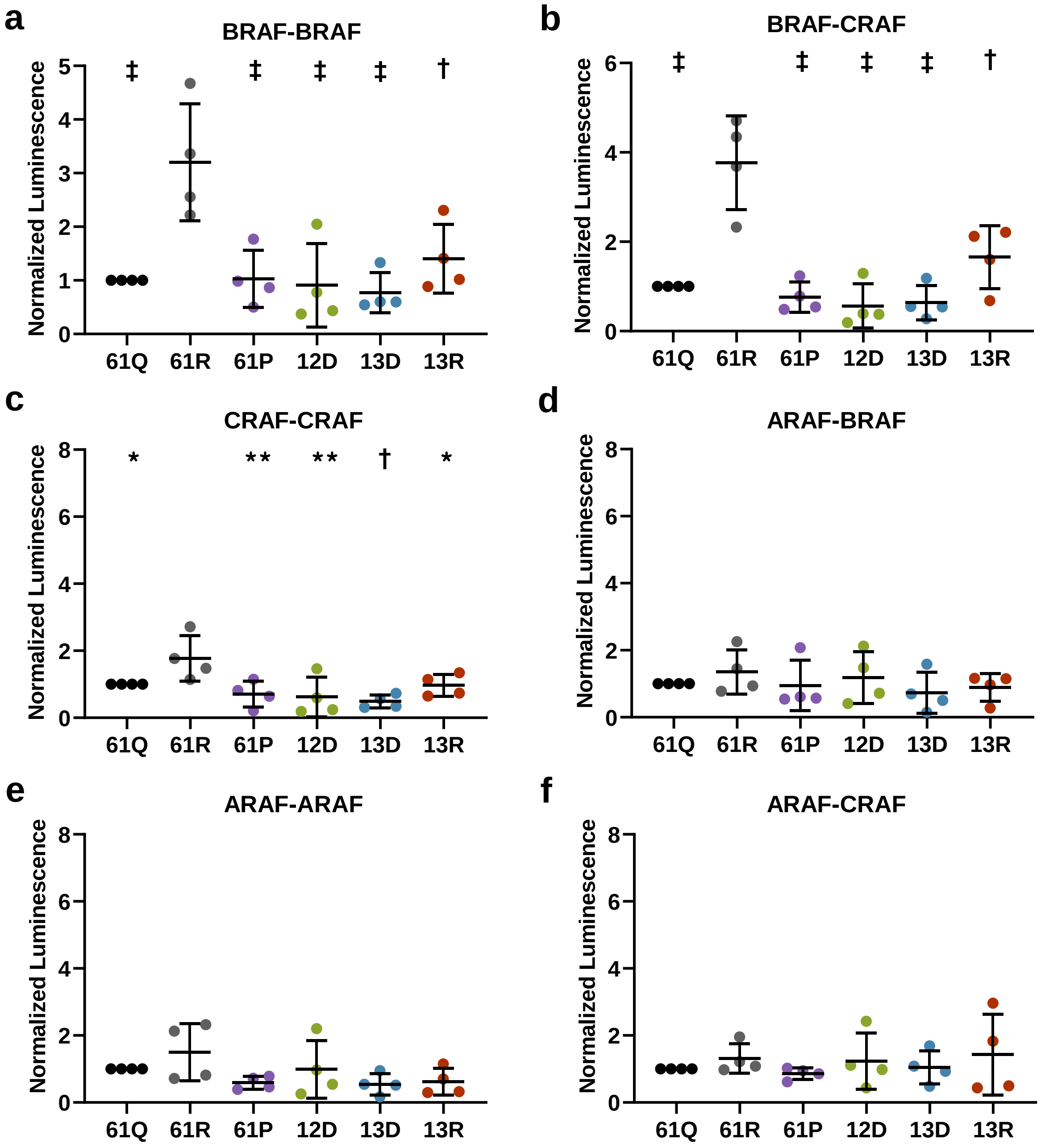

Supplementary Figure 11: NRAS codon 12 and 13 mutants do not enhance RAF dimerization. a-f, Dot plots of normalized luminescence intensity in *TN*$^{X/X}$ MEFs infected with adenovirus expressing BRAF-LgBiT and BRAF-SmBiT (**a**), BRAF-LgBiT and Craf-SmBiT (**b**), CRAF-LgBiT and CRAF-SmBiT (**c**), ARAF-LgBiT and BRAF-SmBiT (**d**), ARAF-LgBiT and ARAF-SmBiT (**e**) or ARAF-LgBiT and CRAF-SmBiT (**f**). Luminescence intensity was normalized to crystal violet staining for each well. Dot plot data are presented as mean values +/- SD where each dot represents one biological replicate. n = 4 biologically independent replicates per genotype were examined over 4 independent experiments. One-way ANOVA with a Tukey's post-test was used to compare data between each genotype. NRAS mutant samples statistically different from NRAS$^{61R/R}$ samples are indicated in the figure. Adjusted p-values for all comparisons can be found in Supplementary Table 4e. * p< 0.05, ** p< 0.01, † p< 0.001, ‡ p< 0.0001. Source data are provided as a Source Data file.

a **Q61R**

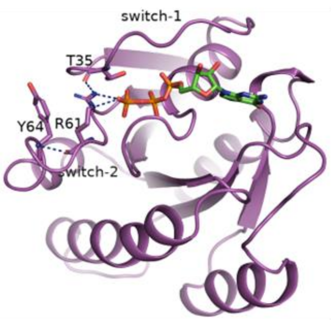

Cluster 1

Interactions	Type	Dist (Å)
R61 (NH2) – T35 (O)	H-bond	2.8
R61 (NH2) – GTP (O2G)	H-bond	2.7
R61 (NE) – GTP (O2G)	H-bond	2.9
R61 (O) – Y64 (N)	H-bond	2.9

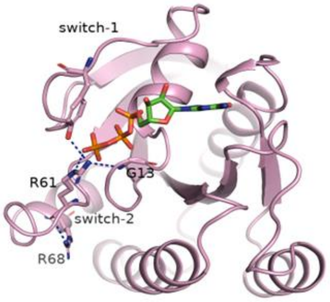

Cluster 2

Interactions	Type	Dist (Å)
R61 (NH2) – Y32 (OH)	H-bond	3.3
R61 (NH2) – G13 (N)	H-bond	3.5
R61 (NE) – GTP (O2G)	H-bond	3.4
R61 (O) – R68 (NH2)	H-bond	3.2

b **Q61K**

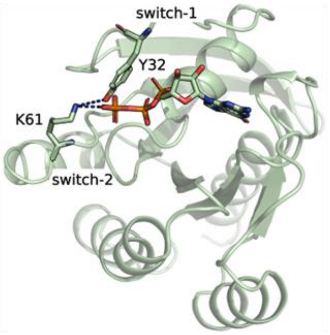

Cluster 1

Interactions	Type	Dist (Å)
K61 (NZ) – Y32 (OH)	H-bond	3.0
K61 (NZ) – GTP (O2G)	H-bond	3.0

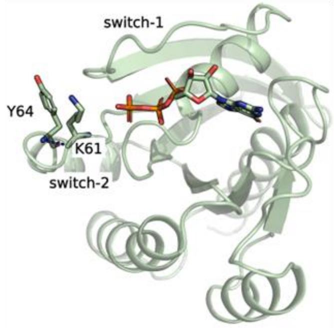

Cluster 2

Interactions	Type	Dist (Å)
K61 (O) – Y64 (N)	H-bond	3.2

c **Q61L**

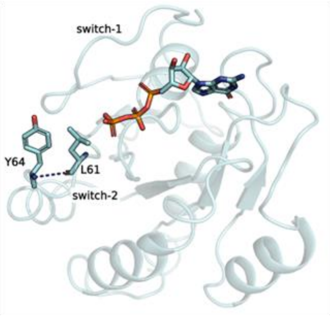

Cluster 1

Interactions	Type	Dist (Å)
L61 (O) – Y64 (N)	H-bond	3.3

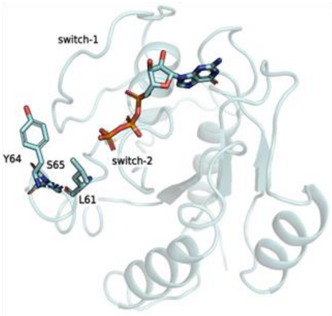

Cluster 2

Interactions	Type	Dist (Å)
L61 (O) – Y64 (N)	H-bond	3.1
L61 (O) – S65 (N)	H-bond	2.9

d **Q61H**

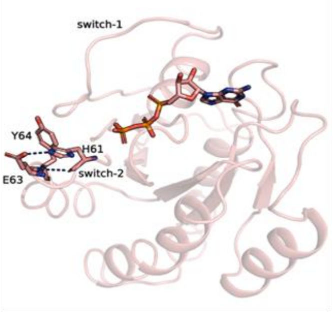

Cluster 1

Interactions	Type	Dist (Å)
H61 (NE2) – E63 (OE2)	H-bond	2.8
H61 (O) – Y64 (NH1)	H-bond	3.2

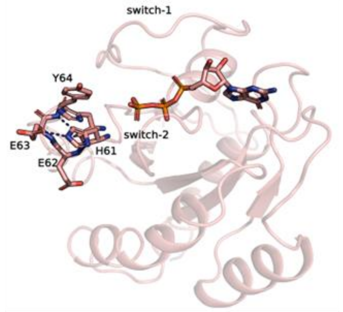

Cluster 2

Interactions	Type	Dist (Å)
H61 (ND1) – E62 (N)	H-bond	3.4
H61 (NE) – E63 (OE1)	H-bond	2.8
H61 (O) – Y64 (N)	H-bond	3.4

e **Q61P**

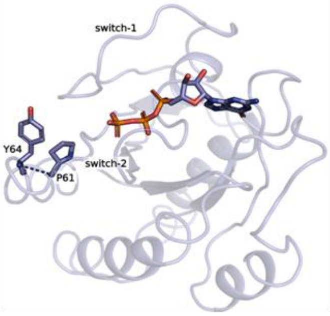

Cluster 1

Interactions	Type	Dist (Å)
P61 (O) – Y64 (N)	H-bond	3.4

f

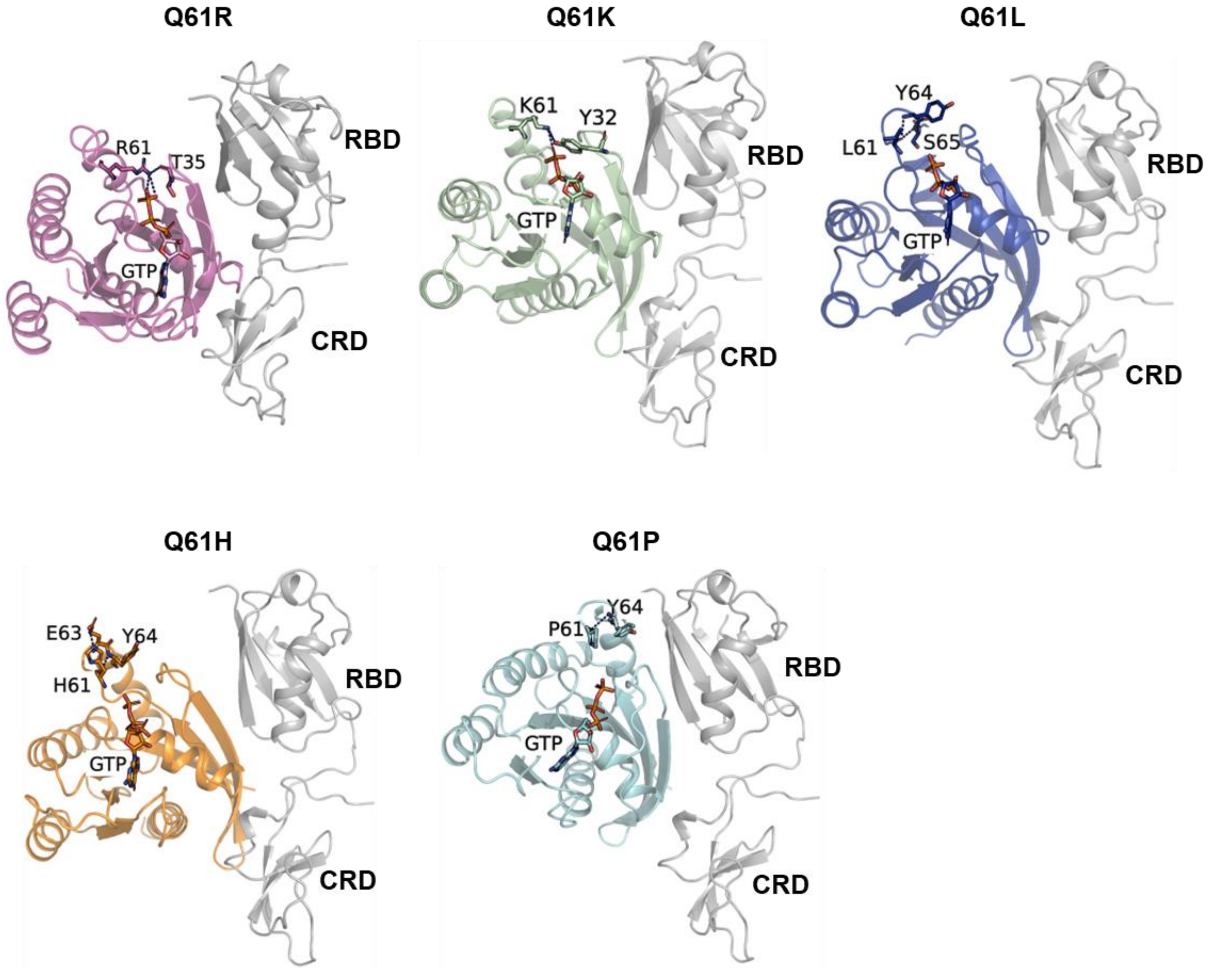

Supplementary Figure 12: Conformational changes induced by the codon 61 mutant alter affinity for the BRAF RBD-CRD. a-e, Representative conformations of NRAS codon 61 mutants extracted from highly populated REMD structural ensembles. Intramolecular interactions with the codon 61 sidechain are listed below each structure. **f**, Molecular docking of NRAS codon 61 mutants with the BRAF-RBDCRD. The binding orientations of NRAS mutants were estimated through molecular docking calculations. Average conformations of NRAS codon 61 mutants were extracted from replica-exchange molecular dynamics (REMD) simulation trajectories and subsequently docked against BRAF-RBDCRD using the Hex docking program. The differential interactions of NRAS codon 61 mutants with BRAF mirror their distinct biochemical and downstream signaling profiles in melanoma. NRAS codon 61 mutants and BRAF-RBDCRD are shown in cartoon representation and bound nucleotide in licorice. The BRAF-RBDCRD is gray and polar interactions between the mutant amino acid and surrounding residues are indicated with a blue dashed line.

Supplementary Tables

Supplementary Table 1a: Comparison of melanoma kinetics and burden in homozygous *TN*^{61X} models

LSL-Nras^{61X} Genotype	Median MFS	Median OS	Mean TB (Melanomas / Mouse)	Mean TG log_{10}(% Tumor Volume / Wk)
**Q/Q vs. R/R**	**<0.0001‡**	**<0.0001‡**	N/A	N/A
**Q/Q vs. K/K**	**<0.0001‡**	**<0.0001‡**	N/A	N/A
**Q/Q vs. L/L**	**<0.0001‡**	**<0.0001‡**	N/A	N/A
**Q/Q vs. H/H**	**<0.0001‡**	**<0.0001‡**	N/A	N/A
**Q/Q vs. P/P**	>0.9999	0.7168	N/A	N/A
**R/R vs. K/K**	0.9553	0.6507	0.3002	0.9973
**R/R vs. L/L**	**0.0465***	**0.0071****	**0.0020****	0.2985
**R/R vs. H/H**	**<0.0001‡**	**<0.0001‡**	**<0.0001‡**	0.9379
**R/R vs. P/P**	**<0.0001‡**	**<0.0001‡**	N/A	N/A
**K/K vs. L/L**	**0.0209***	**0.0043****	**0.0019****	0.4793
**K/K vs. H/H**	**<0.0001‡**	**<0.0001‡**	**<0.0001‡**	0.972
**K/K vs. P/P**	**<0.0001‡**	**<0.0001‡**	N/A	N/A
**L/L vs. H/H**	**0.0015****	**0.0010****	**0.0059****	0.9194
**L/L vs. P/P**	**<0.0001‡**	**<0.0001‡**	N/A	N/A
**H/H vs. P/P**	**<0.0001‡**	**<0.0001‡**	N/A	N/A

* $p < 0.05$, ** $p < 0.01$, ‡ $p < 0.001$

	F value	Degrees of Freedom (DFn, DFd)
**Mean TB**	36.43	(3.000, 42.81)
**Mean TG**	1.086	(3, 410)

Supplementary Table 1b: Comparison of melanoma kinetics and burden in *TN^61R/R^* mice from each experimental cohort.

LSL-Nras^{61X} Genotype	Median MFS	Median OS	Mean TB (Melanomas / Mouse)	Mean TG $\log_{10}$(% Tumor Volume / Wk)
**R/R (Q) vs. R/R (K)**	0.7567	0.4126	0.8993	0.9943
**R/R (Q) vs. R/R (L)**	0.7005	0.6966	0.3512	0.9748
**R/R (Q) vs. R/R (H)**	0.8433	0.9098	0.7361	0.9912
**R/R (Q) vs. R/R (P)**	0.8614	0.3894	0.7909	0.5935
**R/R (K) vs. R/R (L)**	0.5174	0.8284	0.9229	0.8878
**R/R (K) vs. R/R (H)**	0.9993	0.5076	0.9997	>0.9999
**R/R (K) vs. R/R (P)**	0.9624	0.9694	0.9998	0.4582
**R/R (L) vs. R/R (H)**	0.5894	0.7624	0.9565	0.8266
**R/R (L) vs. R/R (P)**	0.4996	0.6893	0.9584	0.9176
**R/R (H) vs. R/R (P)**	0.8925	0.1915	>0.9999	0.3038

	F value	Degrees of Freedom (DFn, DFd)
**Mean TB**	0.9502	(4, 67)
**Mean TG**	1.218	(4, 247)

Supplementary Table 1c: Comparison of melanoma-free survival between male and female *TN*$^{61R/R}$ mice

Sex	Median MFS
**Male vs. Female**	0.4597

Supplementary Table 1d: ANOVA analysis of IHC staining quantification in murine melanomas expressing the denoted NRAS codon 61 mutants.

Genotype	Ki67	CD45	Caspase 3
**R/R vs. K/K**	0.9854	0.6851	0.9986
**R/R vs. L/L**	0.9869	0.6445	0.9947
**R/R vs. H/H**	**0.0329***	0.9864	0.8699

* $p < 0.05$

	Ki67	CD45	Caspase 3
**F value**	4.198	0.8218	0.142
**Degrees of Freedom (DFn, DFd)**	(3, 11)	(3, 13)	(3, 11)
**R/R (n)**	4	3	4
**K/K (n)**	4	6	4
**L/L (n)**	4	4	4
**H/H (n)**	3	4	3

Supplementary Table 1e: Melanoma kinetics and burden in $TN^{61X/X}$ models treated with or without UVB.

Cohort	LSL-$Nras^{61X}$ Genotype	Median MFS	Median OS	Mean TB (Melanomas / Mouse)	Mean TG log_{10}(% Tumor Volume / Wk)
**UVB**	**R/R vs K/K**	0.4203	**0.0013****	>0.9999	0.9097
	**R/R vs L/L**	**0.0250***	**<0.0001‡**	0.5093	0.1601
	**R/R vs H/H**	**<0.0001‡**	**<0.0001‡**	0.1400	**0.0087****
	**K/K vs L/L**	0.6412	0.0852	0.6102	0.8083
	**K/K vs H/H**	**0.0076****	**0.0073****	0.1541	0.1691
	**L/L vs H/H**	**0.0452***	0.0687	0.9997	0.9627
**No UVB**	**R/R vs K/K**	0.1413	0.2328	0.8027	0.9515
	**R/R vs L/L**	**0.0278***	0.1502	0.9683	0.9985
	**R/R vs H/H**	**0.0018****	**0.0114***	0.8027	0.9970
	**K/K vs L/L**	0.5114	0.8615	0.5892	>0.9999
	**K/K vs H/H**	0.0581	0.1275	0.9991	>0.9999
	**L/L vs H/H**	0.1621	0.1561	0.5892	>0.9999
**UVB vs No UVB**	**R/R**	**<0.0001‡**	**<0.0001‡**	**0.0110***	>0.9999
	**K/K**	**<0.0001‡**	**<0.0001‡**	**0.0050****	0.9935
	**L/L**	**<0.0001‡**	**0.0003†**	0.6332	0.9983
	**H/H**	**0.002†**	**0.0023****	0.0668	0.9993

***** p< 0.05, ****** p< 0.01, **†** p< 0.001, **‡** p< 0.001

	F value	Degrees of Freedom (DFn, DFd)
**Mean TB**	9.581	(7.000, 22.14)
**Mean TG**	2.461	(7, 205)

Supplementary Table 1f: Summary of melanoma kinetics and burden in *$TN^{61X/X}$* models.

Cohort	Genotype	Median MFS	Median OS	Mean TB (Melanomas / Mouse)	Mean TG log_{10}(% Tumor Volume / Wk)
**61K**	**K/K (n=19)**	6.00	12.71	5.89	0.84
	**K/R (n=19)**	5.57	12.57	5.84	0.76
	**R/R (n=12)**	5.07	12.57	4.36	0.78
**61L**	**L/L (n=17)**	9.00	16.14	**2.71****	0.72
	**L/R (n=17)**	5.14	15.71	**3.14***	0.71
	**R/R (n=13)**	5.57	12.86	5.25	0.88
**61H**	**H/H (n=17)**	**20.71‡**	**27.85‡**	**1.44‡**	0.79
	**H/R (n=16)**	5.43	**18.93****	**2.79***	0.76
	**R/R (n=16)**	5.71	12.28	4.56	0.79
**61P**	**P/P (n=17)**	**>60.00‡**	**>60.00‡**	0.00	N/A
	**P/R (n=18)**	**56.71‡**	**59.85‡**	**1.08****	0.84
	**R/R (n=14)**	5.36	12.43	4.54	0.95
**61Q**	**Q/Q (n=12)**	**>60.00‡**	**>60.00‡**	0.00	N/A
	**Q/R (n=19)**	**>60.00‡**	**>60.00‡**	1.00	0.83
	**R/R (n=20)**	5.86	11.42	3.53	0.82
**12D**	**D/D (n=22)**	>60.00	>60.00	0.00	0.00
**13D**	**D/D (n=23)**	>55.57	>60.00	0.00	0.00
**13R**	**R/R (n=25)**	43.71	>60.00	1.24	0.86

* p< 0.05, ** p< 0.01, ‡ p< 0.001

		F value	Degrees of Freedom (DFn, DFd)
**61K**	**Mean TB**	1.162	(2, 46)
	**Mean TG**	0.7368	(2, 209)
**61L**	**Mean TB**	6.237	(2, 40)
	**Mean TG**	1.749	(2, 134)
**61H**	**Mean TB**	13.31	(2, 20.95)
	**Mean TG**	0.05234	(2, 123)
**61P**	**Mean TB**	4.072	12.23
	**Mean TG**	1.117	68
**61Q**	**Mean TB**	N/A	N/A
	**Mean TG**	N/A	N/A
**12/13**	**Mean TB**	N/A	N/A
	**Mean TG**	N/A	N/A

–Unpaired t-test with Welch's correction used to compare 61P data.

Supplementary Table 2: ANOVA analysis of the effects of NRAS codon 61 mutants on cellular proliferation.

Cohort	Genotype	EdU p-adj.
**MEFs**	Q/Q vs. R/R	**0.0007†**
	Q/Q vs. K/K	**<0.0001‡**
	Q/Q vs. L/L	**<0.0001‡**
	Q/Q vs. H/H	**0.0093****
	Q/Q vs. P/P	0.5975
	R/R vs. K/K	0.7617
	R/R vs. L/L	0.546
	R/R vs. H/H	0.8304
	R/R vs. P/P	**0.0221***
	K/K vs. L/L	0.999
	K/K vs. H/H	0.1646
	K/K vs. P/P	**0.0013****
	L/L vs. H/H	0.0846
	L/L vs. P/P	**0.0006†**
	H/H vs. P/P	0.2151
**Cutaneous melanocytes**	K/K vs. L/L	0.1102
	K/K vs. H/H	**0.0406***
	K/K vs. P/P	**0.0429***
	L/L vs. H/H	0.9428
	L/L vs. P/P	0.8846
	H/H vs. P/P	0.9965

* p< 0.05, ** p< 0.01, † p< 0.001, ‡ p< 0.001

MEFs	EdU
**F value**	15.19
**Degrees of Freedom (DFn, DFd)**	(5, 18)
**Q/Q (n)**	n = 4
**R/R (n)**	n = 4
**K/K (n)**	n = 4
**L/L (n)**	n = 4
**H/H (n)**	n = 4
**P/P (n)**	n = 4

Cutaneous melanocytes	EdU
**F value**	4.655
**Degrees of Freedom (DFn, DFd)**	(3, 12)
**R/R (n)**	n = 5
**K/K (n)**	n = 4
**L/L (n)**	n = 4
**H/H (n)**	n = 3

Supplementary Table 3a: ANOVA analysis of the effects of NRAS codon 61 mutants on cellular signaling.

Cohort	Genotype	ERK p-adj.	AKT p-adj.	NRAS p-adj.
MEFs	Q/Q vs. R/R	**<0.0001‡**	0.9993	0.9874
	Q/Q vs. K/K	**<0.0001‡**	0.9999	>0.9999
	Q/Q vs. L/L	**0.0045****	0.9946	>0.9999
	Q/Q vs. H/H	0.5649	0.9998	0.9987
	Q/Q vs. P/P	0.9979	0.9847	0.9997
	R/R vs. K/K	>0.9999	>0.9999	0.9734
	R/R vs. L/L	**0.0391***	>0.9999	0.9969
	R/R vs. H/H	**<0.0001‡**	>0.9999	0.9998
	R/R vs. P/P	**<0.0001‡**	0.9995	0.9993
	K/K vs. L/L	0.0637	0.9998	0.9998
	K/K vs. H/H	**<0.0001‡**	>0.9999	0.9952
	K/K vs. P/P	**<0.0001‡**	0.9987	0.998
	L/L vs. H/H	0.1556	0.9997	0.9999
	L/L vs. P/P	**0.0009†**	>0.9999	>0.9999
	H/H vs. P/P	0.2711	0.9981	>0.9999
Tumor Cells	R/R vs. K/K	0.9034	0.7471	0.9944
	R/R vs. L/L	**0.0050****	>0.9999	0.4632
	R/R vs. H/H	0.0564	0.7777	**0.027***
	K/K vs. L/L	**0.0008†**	0.7449	0.3508
	K/K vs. H/H	**0.0110***	>0.9999	**0.0171***
	L/L vs. H/H	0.7569	0.7756	**0.0067****

* p< 0.05, ** p< 0.01, † p< 0.001, ‡ p< 0.001

MEFs	EdU	ERK	AKT	NRAS
**F value**	15.19	23.67	0.1105	0.1521
**Degrees of Freedom (DFn, DFd)**	(5, 18)	(5, 44)	(5, 44)	(5, 43)
**Q/Q (n)**	n = 4	n = 8	n = 8	n = 8
**R/R (n)**	n = 4	n = 9	n = 9	n = 9
**K/K (n)**	n = 4	n = 7	n = 7	n = 7
**L/L (n)**	n = 4	n = 7	n = 7	n = 7
**H/H (n)**	n = 4	n = 10	n = 10	n = 9
**P/P (n)**	n = 4	n = 9	n = 9	n = 9

Tumor Cells	ERK	AKT	NRAS
**F value**	8.604	0.6418	7.557
**Degrees of Freedom (DFn, DFd)**	(3, 32)	(3, 32)	(3, 32)
**R/R (n)**	n = 9	n = 9	n = 9
**K/K (n)**	n = 9	n = 9	n = 9
**L/L (n)**	n = 9	n = 9	n = 9
**H/H (n)**	n = 9	n = 9	n = 9

Supplementary Table 3b: ANOVA analysis of the effects of NRAS codon 12 and 13 mutants on cellular proliferation and signaling.

Cohort	Genotype	ERK p-adj.	AKT p-adj.	NRAS p-adj.
MEFs	61Q/Q vs. 61R/R	**<0.0001‡**	0.3237	0.8409
	61Q/Q vs. 61P/P	>0.9999	0.2112	0.8852
	61Q/Q vs. 12D/D	>0.9999	0.8068	**0.0117***
	61Q/Q vs. 13D/D	0.9975	0.3367	0.6571
	61Q/Q vs. 13R/R	0.5964	0.5968	**0.0100***
	61R/R vs. 61P/P	**<0.0001‡**	>0.9999	0.2658
	61R/R vs. 12D/D	**<0.0001‡**	0.9582	0.0993
	61R/R vs. 13D/D	**<0.0001‡**	>0.9999	0.1269
	61R/R vs. 13R/R	**0.0008†**	0.9871	0.1015
	61P/P vs. 12D/D	0.9999	0.9145	**0.0015****
	61P/P vs. 13D/D	0.9991	>0.9999	0.9974
	61P/P vs. 13R/R	0.6494	0.9644	**0.0011****
	12D/D vs. 13D/D	0.9918	0.9635	**0.0007†**
	12D/D vs. 13R/R	0.5722	0.9998	>0.9999
	13D/D vs. 13R/R	0.8398	0.9895	**0.0005†**

* p< 0.05, ** p< 0.01, † p< 0.001, ‡ p< 0.001

	ERK	AKT	NRAS
**F value**	14.55	1.592	11.42
**Degrees of Freedom (DFn, DFd)**	(5, 17)	(5, 15)	(5, 17)
**61Q/Q (n)**	n = 4	n = 4	n = 4
**61R/R (n)**	n = 4	n = 3	n = 4
**61P/P (n)**	n = 4	n = 4	n = 4
**12D/D (n)**	n = 3	n = 3	n = 3
**13D/D (n)**	n = 4	n = 3	n = 4
**13R/R (n)**	n = 4	n = 4	n = 4

Supplementary Table 4a: ANOVA analysis of the effects of RAS or SOS1 knockdown on NRAS codon 61 mutant signaling.

Sample 1	Sample 2	ERK p-adj.	AKT p-adj.
Q/Q eGFP	P/P eGFP	0.9867	>0.9999
Q/Q eGFP	R/R eGFP	**0.0003†**	0.9992
Q/Q eGFP	Q/Q NRAS	0.9831	0.9765
Q/Q eGFP	P/P NRAS	0.2385	>0.9999
Q/Q eGFP	R/R NRAS	0.8945	>0.9999
Q/Q eGFP	Q/Q H/KRAS	0.9381	>0.9999
Q/Q eGFP	P/P H/KRAS	**<0.0001‡**	>0.9999
Q/Q eGFP	R/R H/KRAS	**<0.0001‡**	>0.9999
Q/Q eGFP	Q/Q SOS1	0.9987	0.9524
Q/Q eGFP	P/P SOS1	0.9997	>0.9999
Q/Q eGFP	R/R SOS1	**0.0003†**	>0.9999
P/P eGFP	R/R eGFP	**0.0091****	>0.9999
P/P eGFP	Q/Q NRAS	>0.9999	0.8848
P/P eGFP	P/P NRAS	0.82	>0.9999
P/P eGFP	R/R NRAS	>0.9999	0.999
P/P eGFP	Q/Q H/KRAS	>0.9999	0.9996
P/P eGFP	P/P H/KRAS	**0.0002†**	>0.9999
P/P eGFP	R/R H/KRAS	**<0.0001‡**	>0.9999
P/P eGFP	Q/Q SOS1	0.6265	0.7915
P/P eGFP	P/P SOS1	0.7211	>0.9999
P/P eGFP	R/R SOS1	**0.0082****	0.9999
R/R eGFP	Q/Q NRAS	0.1047	0.7009
R/R eGFP	P/P NRAS	0.8681	>0.9999
R/R eGFP	R/R NRAS	0.2261	0.9835
R/R eGFP	Q/Q H/KRAS	0.1753	0.991
R/R eGFP	P/P H/KRAS	0.95	>0.9999
R/R eGFP	R/R H/KRAS	0.5908	>0.9999
R/R eGFP	Q/Q SOS1	**<0.0001‡**	0.5348
R/R eGFP	P/P SOS1	**<0.0001‡**	0.9978
R/R eGFP	R/R SOS1	>0.9999	0.993
Q/Q NRAS	P/P NRAS	0.9718	0.9689
Q/Q NRAS	R/R NRAS	>0.9999	>0.9999
Q/Q NRAS	Q/Q H/KRAS	>0.9999	0.9998
Q/Q NRAS	P/P H/KRAS	**0.0056****	0.9836
Q/Q NRAS	R/R H/KRAS	**0.0014****	0.9868
Q/Q NRAS	Q/Q SOS1	0.7098	>0.9999
Q/Q NRAS	P/P SOS1	0.7814	0.9913
Q/Q NRAS	R/R SOS1	0.0973	0.9965
P/P NRAS	R/R NRAS	0.9972	>0.9999
P/P NRAS	Q/Q H/KRAS	0.9929	>0.9999
P/P NRAS	P/P H/KRAS	0.1934	>0.9999
P/P NRAS	R/R H/KRAS	0.0549	>0.9999
P/P NRAS	Q/Q SOS1	**0.0465***	0.9508
P/P NRAS	P/P SOS1	0.0625	>0.9999
P/P NRAS	R/R SOS1	0.8537	>0.9999
R/R NRAS	Q/Q H/KRAS	>0.9999	>0.9999
R/R NRAS	P/P H/KRAS	**0.0153***	>0.9999
R/R NRAS	R/R H/KRAS	**0.0037****	>0.9999
R/R NRAS	Q/Q SOS1	0.4586	>0.9999
R/R NRAS	P/P SOS1	0.5371	>0.9999
R/R NRAS	R/R SOS1	0.2126	>0.9999
Q/Q H/KRAS	P/P H/KRAS	**0.0108***	>0.9999
Q/Q H/KRAS	R/R H/KRAS	**0.0026****	>0.9999
Q/Q H/KRAS	Q/Q SOS1	0.5464	0.9997
Q/Q H/KRAS	P/P SOS1	0.6264	>0.9999
Q/Q H/KRAS	R/R SOS1	0.1641	>0.9999
P/P H/KRAS	R/R H/KRAS	0.9996	>0.9999
P/P H/KRAS	Q/Q SOS1	**<0.0001‡**	0.9739
P/P H/KRAS	P/P SOS1	**<0.0001‡**	>0.9999
P/P H/KRAS	R/R SOS1	0.9577	>0.9999
R/R H/KRAS	Q/Q SOS1	**<0.0001‡**	0.979
R/R H/KRAS	P/P SOS1	**<0.0001‡**	>0.9999
R/R H/KRAS	R/R SOS1	0.6122	>0.9999
Q/Q SOS1	P/P SOS1	>0.9999	0.9834
Q/Q SOS1	R/R SOS1	**<0.0001‡**	0.9934
P/P SOS1	R/R SOS1	**<0.0001‡**	>0.9999

* p< 0.05, ** p< 0.01, † p< 0.001, ‡ p< 0.001

		ERK	AKT
	**F value**	14.06	0.7781
	**Degrees of Freedom (DFn, DFd)**	(11, 41)	(11, 39)
**eGFP**	**Q/Q (n)**	n = 6	n = 6
	**P/P (n)**	n = 6	n = 6
	**R/R (n)**	n = 5	n = 6
**NRAS**	**Q/Q (n)**	n = 3	n = 3
	**P/P (n)**	n = 3	n = 3
	**R/R (n)**	n = 3	n = 3
**H/KRAS**	**Q/Q (n)**	n = 3	n = 3
	**P/P (n)**	n = 4	n = 3
	**R/R (n)**	n = 3	n = 3
**SOS1**	**Q/Q (n)**	n = 6	n = 5
	**P/P (n)**	n = 6	n = 5
	**R/R (n)**	n = 5	n = 5

Supplementary Table 4b: ANOVA analysis of the effects of RAF knockdown on NRAS codon 61 mutant signaling.

Sample 1	Sample 2	ERK p-adj.	AKT p-adj.
Q/Q eGFP	P/P eGFP	0.2796	0.3157
Q/Q eGFP	R/R eGFP	0.0004†	0.813
Q/Q eGFP	Q/Q ARAF	0.5468	0.244
Q/Q eGFP	P/P ARAF	0.0694	0.7136
Q/Q eGFP	R/R ARAF	0.0400*	0.3521
Q/Q eGFP	Q/Q BRAF	0.9899	0.998
Q/Q eGFP	P/P BRAF	0.9985	0.0293*
Q/Q eGFP	R/R BRAF	0.0456*	0.0235*
Q/Q eGFP	Q/Q CRAF	0.9673	0.3255
Q/Q eGFP	P/P CRAF	>0.9999	0.3075
Q/Q eGFP	R/R CRAF	0.0392*	0.7572
P/P eGFP	R/R eGFP	0.0007†	>0.9999
P/P eGFP	Q/Q ARAF	>0.9999	0.0649
P/P eGFP	P/P ARAF	0.9686	0.1443
P/P eGFP	R/R ARAF	0.0578	>0.9999
P/P eGFP	Q/Q BRAF	0.3736	>0.9999
P/P eGFP	P/P BRAF	0.4116	0.557
P/P eGFP	R/R BRAF	0.6177	0.2478
P/P eGFP	Q/Q CRAF	0.1362	0.1228
P/P eGFP	P/P CRAF	0.864	0.1225
P/P eGFP	R/R CRAF	0.9771	0.4497
R/R eGFP	Q/Q ARAF	0.0012**	0.0732
R/R eGFP	P/P ARAF	0.0363*	0.3588
R/R eGFP	R/R ARAF	0.6435	>0.9999
R/R eGFP	Q/Q BRAF	0.0002†	>0.9999
R/R eGFP	P/P BRAF	<0.0001‡	>0.9999
R/R eGFP	R/R BRAF	0.0746	0.9969
R/R eGFP	Q/Q CRAF	<0.0001‡	0.0949
R/R eGFP	P/P CRAF	0.0004†	0.0951
R/R eGFP	R/R CRAF	0.006**	0.3901
Q/Q ARAF	P/P ARAF	0.8213	0.9255
Q/Q ARAF	R/R ARAF	0.0557	0.0342*
Q/Q ARAF	Q/Q BRAF	0.5066	0.1921
Q/Q ARAF	P/P BRAF	0.5662	0.011*
Q/Q ARAF	R/R BRAF	0.5713	0.0051**
Q/Q ARAF	Q/Q CRAF	0.2308	>0.9999
Q/Q ARAF	P/P CRAF	0.9501	>0.9999
Q/Q ARAF	R/R CRAF	0.9459	>0.9999
P/P ARAF	R/R ARAF	0.1205	0.1019
P/P ARAF	Q/Q BRAF	0.0506	0.7499
P/P ARAF	P/P BRAF	0.0534	0.0079**
P/P ARAF	R/R BRAF	>0.9999	0.0045**
P/P ARAF	Q/Q CRAF	0.0255*	0.8476
P/P ARAF	P/P CRAF	0.1703	0.7757
P/P ARAF	R/R CRAF	>0.9999	0.9889
R/R ARAF	Q/Q BRAF	0.0241*	>0.9999
R/R ARAF	P/P BRAF	0.025*	0.9998
R/R ARAF	R/R BRAF	0.1432	0.9383
R/R ARAF	Q/Q CRAF	0.0353*	0.0661
R/R ARAF	P/P CRAF	0.0329*	0.0764
R/R ARAF	R/R CRAF	0.1157	0.3348
Q/Q BRAF	P/P BRAF	>0.9999	0.9338
Q/Q BRAF	R/R BRAF	0.0289*	0.7048
Q/Q BRAF	Q/Q CRAF	>0.9999	0.2151
Q/Q BRAF	P/P CRAF	>0.9999	0.203
Q/Q BRAF	R/R CRAF	0.0762	0.5987
P/P BRAF	R/R BRAF	0.0298*	>0.9999
P/P BRAF	Q/Q CRAF	>0.9999	0.0281*
P/P BRAF	P/P CRAF	>0.9999	0.0306*
P/P BRAF	R/R CRAF	0.0682	0.1846
R/R BRAF	Q/Q CRAF	0.0147*	0.0187*
R/R BRAF	P/P CRAF	0.0965	0.0207*
R/R BRAF	R/R CRAF	0.9998	0.1329
Q/Q CRAF	P/P CRAF	>0.9999	>0.9999
Q/Q CRAF	R/R CRAF	0.0088**	>0.9999
P/P CRAF	R/R CRAF	0.2267	>0.9999

* p< 0.05, ** p< 0.01, † p< 0.001

		ERK	AKT
	F value	19.77	10.20
	Degrees of Freedom (DFn, DFd)	(11.00, 24.41)	(11.00, 25.93)
eGFP	Q/Q (n)	n = 8	n = 8
	P/P (n)	n = 8	n = 8
	R/R (n)	n = 8	n = 8
NRAS	Q/Q (n)	n = 6	n = 6
	P/P (n)	n = 6	n = 6
	R/R (n)	n = 6	n = 6
H/KRAS	Q/Q (n)	n = 7	n = 7
	P/P (n)	n = 7	n = 7
	R/R (n)	n = 6	n = 7
SOS1	Q/Q (n)	n = 7	n = 8
	P/P (n)	n = 7	n = 8
	R/R (n)	n = 6	n = 8

Supplementary Table 4c: ANOVA analysis of RAF NanoBiT analysis in primary melanocytes expressing the indicated NRAS codon 61 mutants.

**Genotype**	**BRAF-BRAF p-adj.**	**BRAF-CRAF p-adj.**	**CRAF-CRAF p-adj.**
**Q/Q vs. R/R**	**<0.0001‡**	**<0.0001‡**	**0.0025****
**Q/Q vs. K/K**	**0.0107***	**<0.0001‡**	**<0.0001‡**
**Q/Q vs. P/P**	0.1315	**<0.0001‡**	0.706
**Q/Q vs. L/L**	0.6831	0.9866	0.9846

***** p< 0.05, ****** p< 0.01, **†** p< 0.001, **‡** p< 0.001

	**BRAF-BRAF**	**BRAF-CRAF**	**CRAF-CRAF**
**F value**	9.648	21.88	19.37
**Degrees of Freedom (DFn, DFd)**	(4, 171)	(4, 182)	(4, 174)
**Q/Q (n)**	13	13	13
**R/R (n)**	12	12	11
**K/K (n)**	6	6	6
**L/L (n)**	6	6	6
**P/P (n)**	6	7	6

Supplementary Table 4d: ANOVA analysis of RAF NanoBiT analysis in MEFs expressing the indicated NRAS codon 61 mutants.

Genotype	BRAF-BRAF p-adj.	BRAF-CRAF p-adj.	CRAF-CRAF p-adj.	ARAF-BRAF p-adj.	ARAF-ARAF p-adj.	ARAF-CRAF p-adj.
**Q/Q vs. R/R**	**0.0017****	**<0.0001‡**	0.735	0.5731	0.7651	0.9684
**Q/Q vs. H/H**	0.6730	0.5771	0.9512	0.9212	0.9855	0.95
**Q/Q vs. P/P**	0.9604	>0.9999	0.8893	0.8883	0.8528	0.9997
**R/R vs. H/H**	**0.0248***	**0.0003†**	0.9695	0.2632	0.569	0.9998
**R/R vs. P/P**	**0.0012****	**<0.0001‡**	0.3662	0.231	0.3298	0.9829
**H/H vs. P/P**	0.4397	0.6039	0.6496	0.9997	0.9667	0.9698

* p< 0.05, ** p< 0.01, † p< 0.001, ‡ p< 0.001

	BRAF-BRAF	BRAF-CRAF	CRAF-CRAF	ARAF-BRAF	ARAF-ARAF	ARAF-CRAF
**F value**	10.49	23.75	1.041	1.753	1.137	0.1375
**Degrees of Freedom (DFn, DFd)**	(3, 14)	(3, 15)	(3, 14)	(3, 12)	(3, 12)	(3, 12)
**Q/Q (n)**	n = 5	n = 5	n = 5	n = 4	n = 4	n = 4
**R/R (n)**	n = 5	n = 5	n = 5	n = 4	n = 4	n = 4
**H/H (n)**	n = 4	n = 4	n = 4	n = 4	n = 4	n = 4
**P/P (n)**	n = 4	n = 5	n = 4	n = 4	n = 4	n = 4

Supplementary Table 4e: ANOVA analysis of RAF NanoBiT analysis in MEFs expressing the indicated NRAS codon 12 and 13 mutants.

Genotype	BRAF-BRAF p-adj.	BRAF-CRAF p-adj.	CRAF-CRAF p-adj.	ARAF-BRAF p-adj.	ARAF-ARAF p-adj.	ARAF-CRAF p-adj.
61Q/Q vs. 61R/R	<0.0001‡	<0.0001‡	0.0133*	0.9559	0.7838	0.9844
61Q/Q vs. 61P/P	0.9993	0.9997	0.9476	>0.9999	0.8881	0.9996
61Q/Q vs. 12D/D	0.9997	>0.9999	0.9543	0.9979	>0.9999	0.9953
61Q/Q vs. 13D/D	0.9932	0.9883	0.8525	0.9874	0.8357	>0.9999
61Q/Q vs. 13R/R	0.9472	0.8401	>0.9999	0.9998	0.9123	0.9353
61R/R vs. 61P/P	<0.0001‡	<0.0001‡	0.0023**	0.9247	0.2211	0.9257
61R/R vs. 12D/D	<0.0001‡	<0.0001‡	0.0025**	0.9982	0.7733	>0.9999
61R/R vs. 13D/D	<0.0001‡	<0.0001‡	0.0008†	0.6883	0.1801	0.9925
61R/R vs. 13R/R	<0.0001‡	0.0001†	0.0135*	0.876	0.2464	0.9998
61P/P vs. 12D/D	>0.9999	>0.9999	>0.9999	0.9931	0.8956	0.9631
61P/P vs. 13D/D	0.9431	0.9385	>0.9999	0.9953	>0.9999	0.9985
61P/P vs. 13R/R	0.9941	0.9457	0.9461	>0.9999	>0.9999	0.8209
12D/D vs. 13D/D	0.9568	0.9736	0.9998	0.8925	0.8448	0.9983
12D/D vs. 13R/R	0.9905	0.8933	0.9529	0.9812	0.9188	0.9981
13D/D vs. 13R/R	0.7039	0.4758	0.8496	0.9989	>0.9999	0.9591

	BRAF-BRAF	BRAF-CRAF	CRAF-CRAF	ARAF-BRAF	ARAF-ARAF	ARAF-CRAF
F value	13.2	13.02	5.905	0.5403	1.831	0.4184
Degrees of Freedom (DFn, DFd)	(5, 30)	(5, 29)	(5,27)	(5, 18)	(5, 18)	(5, 18)
61Q/Q (n)	n = 6	n = 6	n = 6	n = 4	n = 4	n = 4
61R/R (n)	n = 6	n = 5	n = 5	n = 4	n = 4	n = 4
61P/P (n)	n = 6	n = 6	n = 5	n = 4	n = 4	n = 4
12D/D (n)	n = 6	n = 6	n = 5	n = 4	n = 4	n = 4
13D/D (n)	n = 6	n = 6	n = 6	n = 4	n = 4	n = 4
13R/R (n)	n = 6	n = 6	n = 6	n = 4	n = 4	n = 4

Supplementary Table 5a: Oligoucleotides used to generate and screen *TN* cohorts.

Genotype	Homology Oligos
**Q61K**	TCACTCTTTCATATTCCCAGGATTCTTACCGAAAGCAAGTGGTGATTGATGGTGAGACCTGCTTGCT GGACATACTGGACACAGCTGGAAAGGAGGAGTACAGTGCCATGAGAGACCAGTACATGAGGACA GGCGAAGGGTTCCTCTGTGTATTTGCCATCAATAATAGCAAATCATTTG
**Q61H**	TCACTCTTTCATATTCCCAGGATTCTTACCGAAAGCAAGTGGTGATTGATGGTGAGACCTGCTTGCT GGACATACTGGACACAGCTGGACATGAGGAGTACAGTGCCATGAGAGACCAGTACATGAGGACA GGCGAAGGGTTCCTCTGTGTATTTGCCATCAATAATAGCAAATCATTTG
**Q61L**	TCACTCTTTCATATTCCCAGGATTCTTACCGAAAGCAAGTGGTGATTGATGGTGAGACCTGCTTGCT GGACATACTGGACACAGCTGGACTGGAGGAGTACAGTGCCATGAGAGACCAGTACATGAGGACA GGCGAAGGGTTCCTCTGTGTATTTGCCATCAATAATAGCAAATCATTTG
**Q61P**	TCACTCTTTCATATTCCCAGGATTCTTACCGAAAGCAAGTGGTGATTGATGGTGAGACCTGCTTGCT GGACATACTGGACACAGCTGGACCTGAGGAGTACAGTGCCATGAGAGACCAGTACATGAGGACA GGCGAAGGGTTCCTCTGTGTATTTGCCATCAATAATAGCAAATCATTTG
**Q61Q**	TCACTCTTTCATATTCCCAGGATTCTTACCGAAAGCAAGTGGTGATTGATGGTGAGACCTGCTTGCT GGACATACTGGACACAGCTGGACAAGAGGAGTACAGTGCCATGAGAGACCAGTACATGAGGACA GGCGAAGGGTTCCTCTGTGTATTTGCCATCAATAATAGCAAATCATTTG
**G12C**	GTTTGCCAGTTTAATCGTAATTGCTGCTTTTCTACAGGTTTTTGCTGGTGTGAAATGACTGAGTACA AGTTGGTGGTGGTTGGAGCATGCGGTGTTGGGAAAAGCGCCTTGACGATCCAGCTAATCCAGAAC CACTTTGTGGATGAATATGATCCCACCATAGAGGTGAGGCCCAGCCCC
**G12D**	GTTTGCCAGTTTAATCGTAATTGCTGCTTTTCTACAGGTTTTTGCTGGTGTGAAATGACTGAGTACA AGTTGGTGGTGGTTGGAGCAGATGGTGTTGGGAAAAGCGCCTTGACGATCCAGCTAATCCAGAAC CACTTTGTGGATGAATATGATCCCACCATAGAGGTGAGGCCCAGCCCC
**G13D**	GTTTGCCAGTTTAATCGTAATTGCTGCTTTTCTACAGGTTTTTGCTGGTGTGAAATGACTGAGTACA AGTTGGTGGTGGTTGGAGCAGGTGATGTTGGGAAAAGCGCCTTGACGATCCAGCTAATCCAGAAC CACTTTGTGGATGAATATGATCCCACCATAGAGGTGAGGCCCAGCCCC
**G13R**	GTTTGCCAGTTTAATCGTAATTGCTGCTTTTCTACAGGTTTTTGCTGGTGTGAAATGACTGAGTACA AGTTGGTGGTGGTTGGAGCAGGTCGGGTTGGGAAAAGCGCCTTGACGATCCAGCTAATCCAGAAC CACTTTGTGGATGAATATGATCCCACCATAGAGGTGAGGCCCAGCCCC

Codon	gRNA(PAM)
**Q61**	ACTGGACACAGCTGGACGAG(AGG)
**G12 / G13**	TGGTTGGAGCAGGTGGTGTT(GGG)

PCR Primer	Sequence
**Q61 (F)**	CACATAGGTAGGCGTATTCATCCTGG
**Q61 (R)**	GGCACATATCTGCTGTTGCACTTG
**G12 / G13 (F)**	CACATAGGTAGGCGTATTCATCCTGG
**G12 / G13 (R)**	GGCACATATCTGCTGTTGCACTTG

Supplementary Table 5b: Mission shRNAs used in knockdown experiments.

Targets	TRCN #	Target Sequence
**ARAF**	TRCN0000022610	CAGGCTCATCAAAGGAAGAAA
**BRAF**	TRCN0000287177	CCGTTGTCAAACATGTGGTTA
**CRAF**	TRCN0000312820	GCTTTGGTACTACAGAACTTT
**NRAS**	TRCN0000034390	CGATGGCACTCAAGGTTGTAT
**HRAS**	TRCN0000034382	CGGGTGAAAGATTCAGATGAT
**KRAS**	TRCN0000301869	CAAGTAGTAATTGATGGAGAA
**SOS1**	TRCN0000065427	CCACCAGGTTTCTGTTTACAT

Supplementary Table 5c: SYBR primers for validation of RNA-sequencing results.

Targets	Primer	Sequence
**Dusp6**	Forward	GCAAATTCCTATCTCGGATCAC
	Reverse	ACCAGGACACCACAGTTT
**Spry2**	Forward	GTTTGCATCAGGACTGGATTTA
	Reverse	CAGGCTTAGAACACATCTGAAC
**Spry4**	Forward	ACTCTGCAGCTCCTCAAA
	Reverse	ATGACTGAGCTGGGATTCA

Source Data

Enhanced BRAF engagement by NRAS mutants capable of promoting melanoma initiation

Murphy *et al.*

Uncropped Images **Page**

Supplementary Figure 1d……………………………...……...………35
Supplementary Figure 1e……………………………...……...………36
Supplementary Figure 2c………………………………………………...37
Supplementary Figure 2d……………………………………………..38
Supplementary Figure 9a…………………………………...………39
Supplementary Figure 10a…………………………………………40

Supplementary Figure 1d

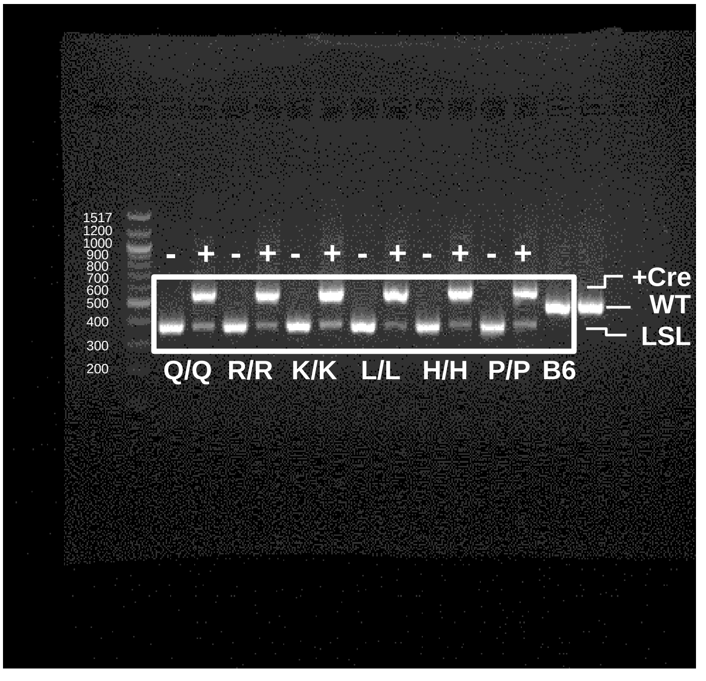

Supplementary Figure 1e

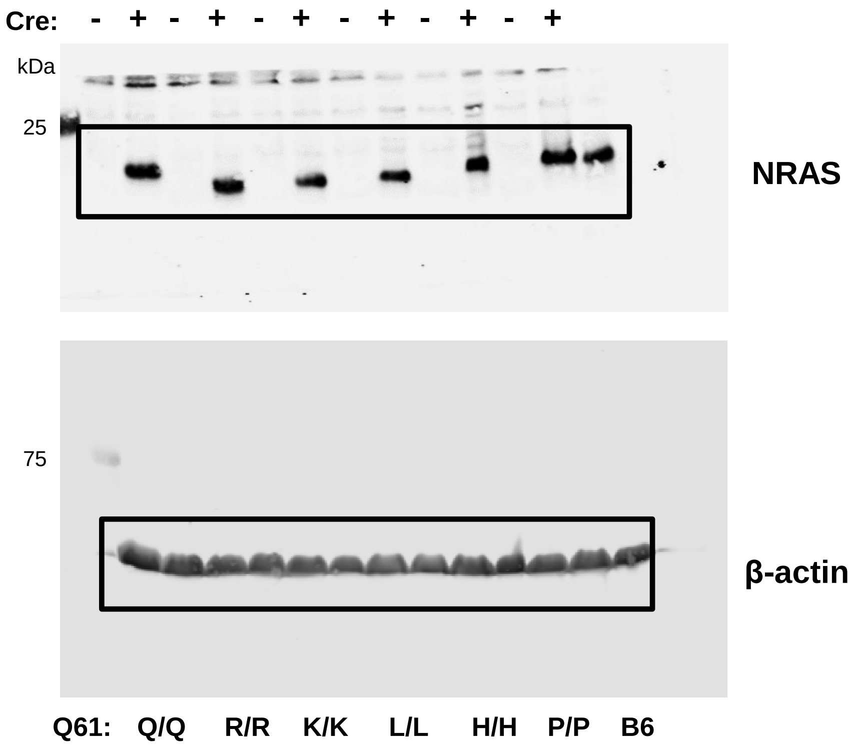

Supplementary Figure 2c

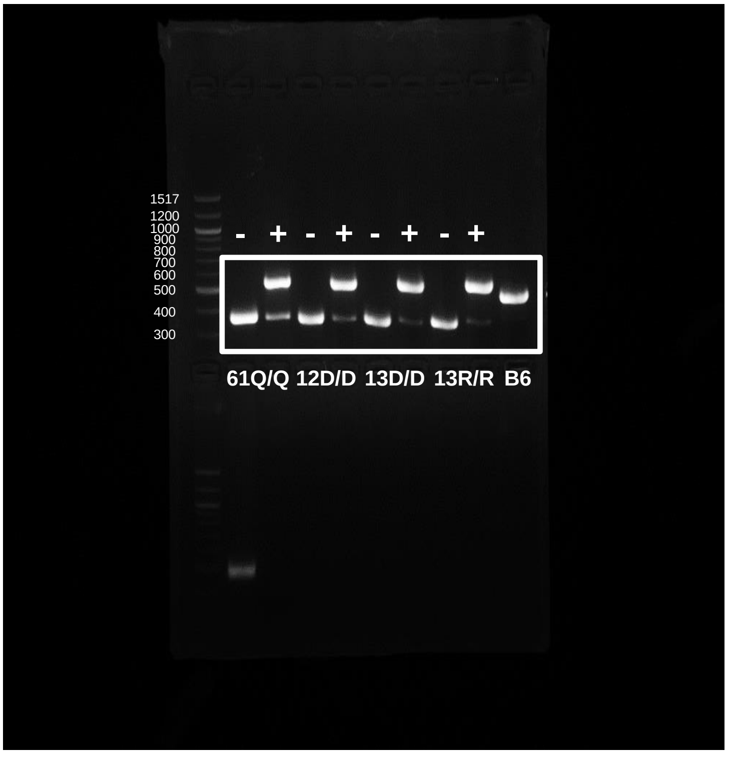

Supplementary Figure 2d

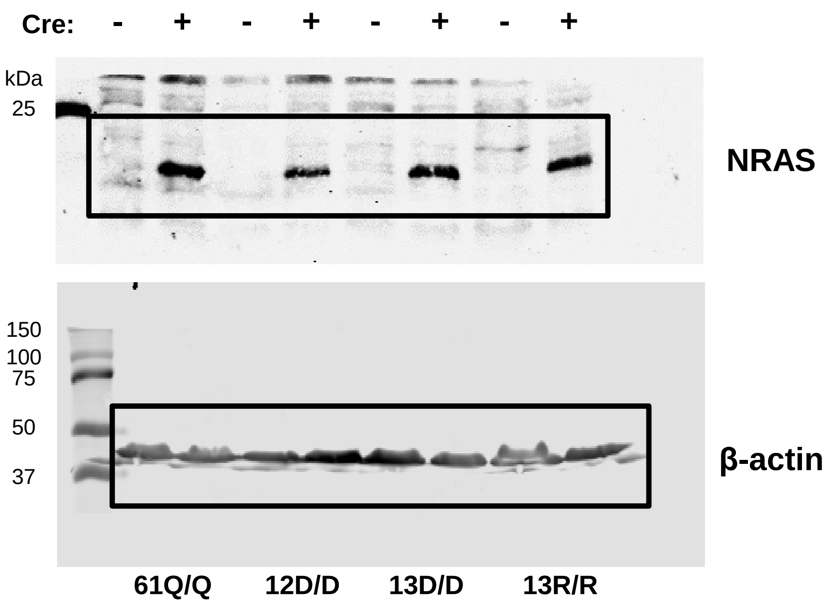

Supplementary Figure 9a

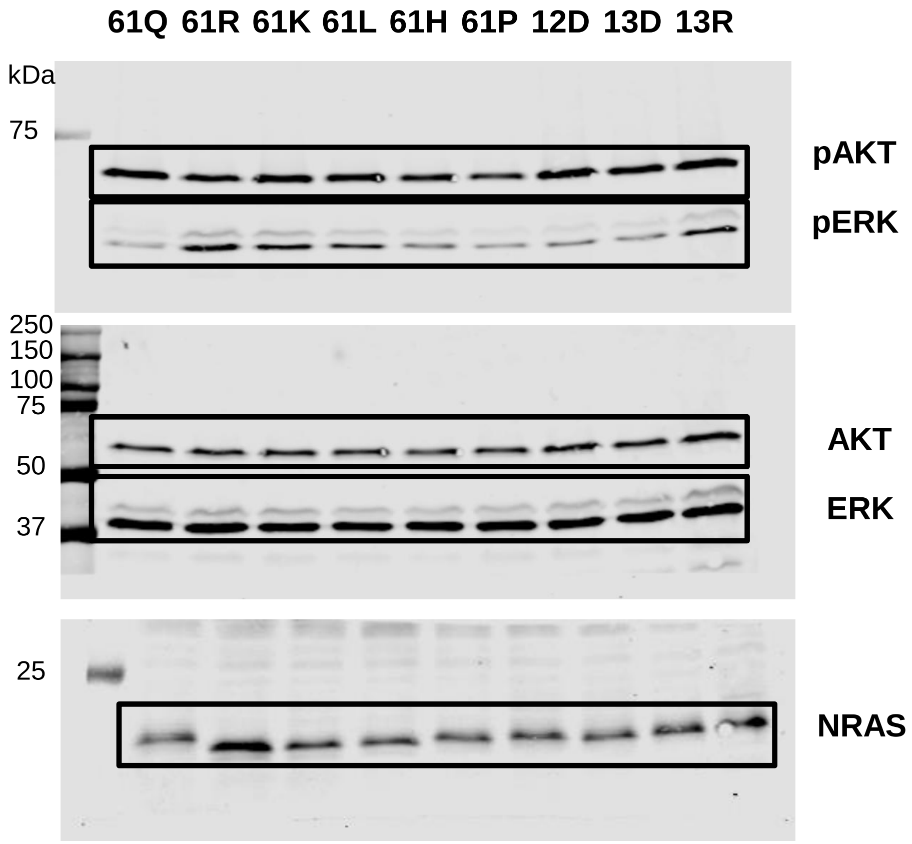

Supplementary Figure 10a

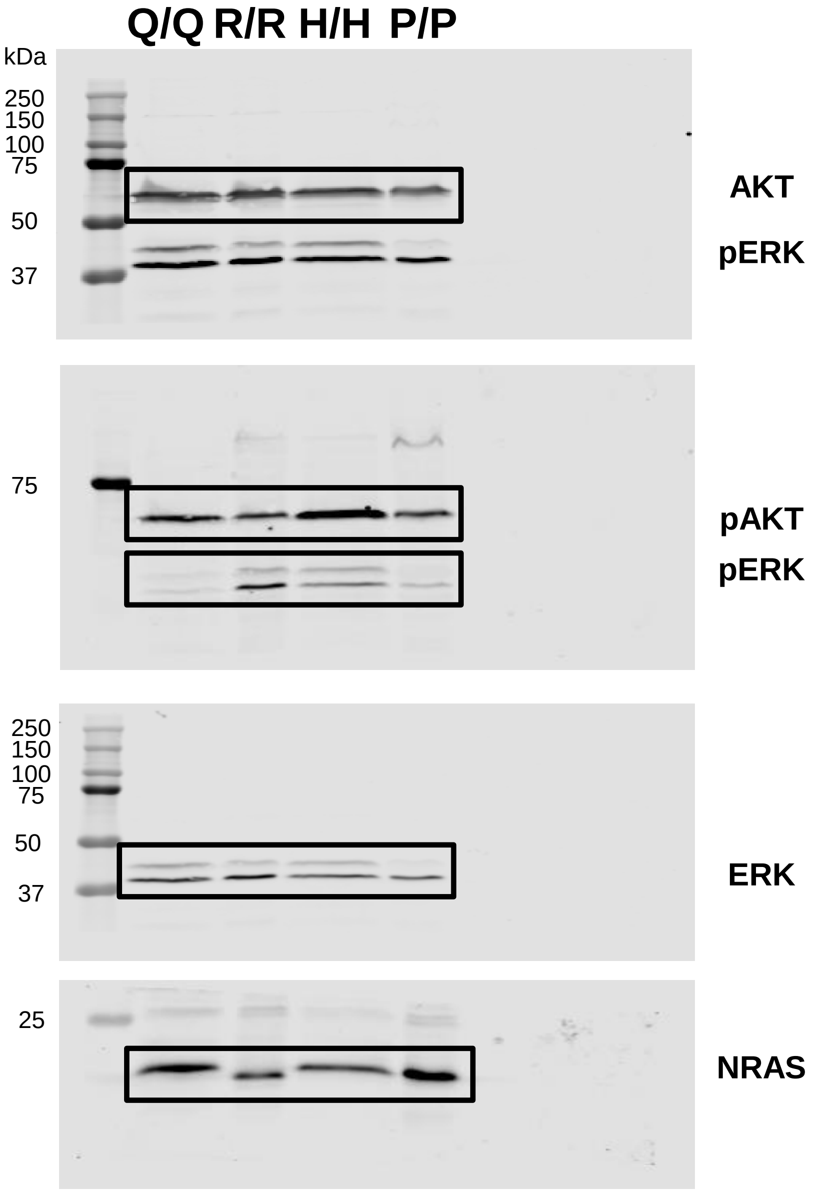

Supplement: Supplementary file 1 — Supplementary Information [file 41467_2022_30881_MOESM1_ESM.pdf]
