# Supplementary material for: Enhanced BRAF engagement by NRAS mutants capable of promoting melanoma initiation
Source: Nat Commun. 2022 Jun 7;13:3153. doi: 10.1038/s41467-022-30881-9 (PMC9174180; doi:10.1038/s41467-022-30881-9)
Supplement: Supplementary file 3 — Description of Additional Supplementary Files [file 41467_2022_30881_MOESM3_ESM.pdf]

Description of Additional Supplementary Files

File Name: Supplementary Data 1
Description: Differential expression analysis of RNA sequencing data between NRAS mutant MEFs and NRAS wild-type MEFs (related to Supplementary Figure 5)

Supplementary Data 1a: DESeq Analysis of MEFs expressing NRAS Q61R versus MEFs expressing NRAS Q61Q (padj < 0.05)

Supplementary Data 1b: DESeq Analysis of MEFs expressing NRAS Q61H versus MEFs expressing NRAS Q61Q (padj < 0.05)

Supplementary Data 1c: DESeq Analysis of MEFs expressing NRAS Q61P versus MEFs expressing NRAS Q61Q (padj < 0.05)

File Name: Supplementary Data 2
Description: Differential expression analysis of RNA sequencing data between NRAS mutant MEFs (related to Figure 3)

Supplementary Data 2a: DESeq Analysis of MEFs expressing NRAS Q61R versus MEFs expressing NRAS Q61H (padj < 0.05)

Supplementary Data 2b: DESeq Analysis of MEFs expressing NRAS Q61R versus MEFs expressing NRAS Q61P (padj < 0.05)

Supplementary Data 2c: DESeq Analysis of MEFs expressing NRAS Q61H versus MEFs expressing NRAS Q61P (padj < 0.05)

File Name: Supplementary Data 3
Description: Differential expression analysis of RNA sequencing data between NRAS mutant murine melanoma samples (related to Supplementary Figure 8)

Supplementary Data 3a: DESeq Analysis of murine melanomas expressing NRAS Q61K versus murine melanomas expressing NRAS Q61R (padj < 0.05)

Supplementary Data 3b: DESeq Analysis of murine melanomas expressing NRAS Q61L versus murine melanomas expressing NRAS Q61R (padj < 0.05

Supplementary Data 3c: DESeq Analysis of murine melanomas expressing NRAS Q61H versus murine melanomas expressing NRAS Q61R (padj < 0.05)
