# Supplementary material for: Enhanced BRAF engagement by NRAS mutants capable of promoting melanoma initiation
Source: Nat Commun. 2022 Jun 7;13:3153. doi: 10.1038/s41467-022-30881-9 (PMC9174180; doi:10.1038/s41467-022-30881-9)
Supplement: Supplementary file 7 — Reporting Summary [file 41467_2022_30881_MOESM7_ESM.pdf]

nature research

Corresponding author(s): Christin E. Burd

Last updated by author(s): May 23, 2022

Reporting Summary

Nature Research wishes to improve the reproducibility of the work that we publish. This form provides structure for consistency and transparency in reporting. For further information on Nature Research policies, see our Editorial Policies and the Editorial Policy Checklist.

Statistics

For all statistical analyses, confirm that the following items are present in the figure legend, table legend, main text, or Methods section.

n/a	Confirmed	
☐	☒	The exact sample size (*n*) for each experimental group/condition, given as a discrete number and unit of measurement
☐	☒	A statement on whether measurements were taken from distinct samples or whether the same sample was measured repeatedly
☐	☒	The statistical test(s) used AND whether they are one- or two-sided<br>*Only common tests should be described solely by name; describe more complex techniques in the Methods section.*
☐	☒	A description of all covariates tested
☐	☒	A description of any assumptions or corrections, such as tests of normality and adjustment for multiple comparisons
☐	☒	A full description of the statistical parameters including central tendency (e.g. means) or other basic estimates (e.g. regression coefficient) AND variation (e.g. standard deviation) or associated estimates of uncertainty (e.g. confidence intervals)
☐	☒	For null hypothesis testing, the test statistic (e.g. *F*, *t*, *r*) with confidence intervals, effect sizes, degrees of freedom and *P* value noted<br>*Give P values as exact values whenever suitable.*
☒	☐	For Bayesian analysis, information on the choice of priors and Markov chain Monte Carlo settings
☒	☐	For hierarchical and complex designs, identification of the appropriate level for tests and full reporting of outcomes
☒	☐	Estimates of effect sizes (e.g. Cohen's *d*, Pearson's *r*), indicating how they were calculated

Our web collection on statistics for biologists contains articles on many of the points above.

Software and code

Policy information about availability of computer code

Data collection	No custom code or software was used.
Data analysis	RNA sequencing reads were aligned to build 38 of the mouse genome (mm10) using STAR (Dobin et al. 2013), duplicates marked using PICARD (version 2.17.11) (http://broadinstitute.github.io/picard/) and a gene count matrix generated by featureCounts (version 1.22.2) (Liao et al. 2014). Differential gene expression analysis was performed using DESeq2 (p-adjusted < 0.05) (Love et al. 2014). Gene set enrichment analysis used the DOSE algorithm within the GSEA function of the clusterProfiler package (Yu et al. 2012; Yu et al. 2015) to probe gene sets from the molecular signatures database Hallmark collection (Liberzon et al. 2015).<br><br>Whole exome sequencing reads were aligned to build 38 of the mouse genome (mm10) using burrows-wheeler aligner (version 0.7.15), duplicates were removed using PICARD (version 2.17.11) (http://broadinstitute.github.io/picard/), and the reads were realigned around indels using GATK version 3.6. Variants were called using Mutect2 51, VarScan2 (version 2.4), and Strelka2. Variants that were not detected by all three algorithms or present in the Ensembl mouse variation database were filtered out of each dataset. Filtered datasets were annotated with Variant Effect Predictor<br><br>For quantification of IHC stained tumor samples, three representative images of each slide were taken on an Olympus BX53M brightfiedl microscope with an SC30 camera attachement. Percentages of DAB and FastRed positive cells were quantified using the ImageJ (version 1.53m) Colocalization Object Counter plugin (version 1.0.0).

For manuscripts utilizing custom algorithms or software that are central to the research but not yet described in published literature, software must be made available to editors and reviewers. We strongly encourage code deposition in a community repository (e.g. GitHub). See the Nature Research guidelines for submitting code & software for further information.

Data

Policy information about availability of data

All manuscripts must include a data availability statement. This statement should provide the following information, where applicable:

- Accession codes, unique identifiers, or web links for publicly available datasets
- A list of figures that have associated raw data
- A description of any restrictions on data availability

The raw RNA sequencing data are available on NCBI Gene Expression Omnibus under the following accession numbers: GSE162124 [https://www.ncbi.nlm.nih.gov/geo/query/acc.cgi?acc=GSE162124] (MEFs) or GSE197841 [https://www.ncbi.nlm.nih.gov/geo/query/acc.cgi?acc=GSE197841] (tumor samples). The raw whole exome sequencing data are available on NCBI Sequencing Read Archive under bioproject number: PRJNA812398 [https://www.ncbi.nlm.nih.gov/bioproject/PRJNA812398/]. RNA sequencing and whole exome sequencing data were aligned to build 38 of the mouse genome (mm10). Data from Supplementary Fig. 4 were obtained from cBioPortal MSKCC Melanoma (cBioPortal for Cancer Genomics: NRAS in Melanoma (MSKCC, Clin Cancer Res 2021)), TCGA PanCancer Atlas (cBioPortal for Cancer Genomics: NRAS in Pan-cancer analysis of whole genomes (ICGC/TCGA, Nature 2020)), and TCGA Cancer Cell Line datasets (cBioPortal for Cancer Genomics: NRAS in Cancer Cell Line Encyclopedia (Novartis/Broad, Nature 2012) and 2 other studies) 37, 38. Data for Supplementary Fig. 6 were obtained from the UCSC Xena platform (https://doi.org/10.1038/s41587-020-0546-8) (UCSC Xena (xenabrowser.net)). The remaining data are available within the Article, Supplementary Information or Source Data file.

Field-specific reporting

Please select the one below that is the best fit for your research. If you are not sure, read the appropriate sections before making your selection.

[x] Life sciences [ ] Behavioural & social sciences [ ] Ecological, evolutionary & environmental sciences

For a reference copy of the document with all sections, see nature.com/documents/nr-reporting-summary-flat.pdf

Life sciences study design

All studies must disclose on these points even when the disclosure is negative.

Sample size	For in vivo studies, power calculations prior to initiating the study determined that a sample size of 15 mice per group would provide 80% power to detect a hazard ratio of 0.28 between TN61R and any single TN61X allele (α = 0.01, adjusting for 5 comparisons, two-sided). For in vitro studies, no sample size calculation was performed prior to the analyses. Using the variability in initial biological replicates from MEF and melanocyte cell lines, we determined that a sample size greater than or equal to 3 would provide enough data points to visualize any trends if present.
Data exclusions	For the in vivo and in vitro dot plots, outliers were detected using a two-sided Grubbs' test in PRISM version 8.4.3.
Replication	All data points in the dot plots from this paper depict biological replicates. Samples sizes for the in vivo studies represent individual mice and the sample sizes for the in vitro studies represent MEF cell lines isolated from separate embryos. All replicates were used unless the data point fulfilled the outlier test. These experiments were replicated in at least 3 independent experiments. All attempts to replicate the data were successful.
Randomization	Mice from each cohort were randomly numbered and blindly monitored three times a week for tumor formation. Internal control (NRAS Q61R/R) mice within each cohort ensured the absence of treatment- or litter-specific biases. Randomization and blinding did not occur when performing in vitro experiments.
Blinding	Mice from each cohort were randomly numbered and blindly monitored three times a week for tumor formation. Investigators were blinded during group allocation, tumor monitoring tumor, data collection and analysis. No randomization or blinding was used for in vitro experiments.

Reporting for specific materials, systems and methods

We require information from authors about some types of materials, experimental systems and methods used in many studies. Here, indicate whether each material, system or method listed is relevant to your study. If you are not sure if a list item applies to your research, read the appropriate section before selecting a response.

Materials & experimental systems

n/a	Involved in the study
☐	☒ Antibodies
☐	☒ Eukaryotic cell lines
☒	☐ Palaeontology and archaeology
☐	☒ Animals and other organisms
☒	☐ Human research participants
☒	☐ Clinical data
☒	☐ Dual use research of concern

Methods

n/a	Involved in the study
☒	☐ ChIP-seq
☐	☒ Flow cytometry
☒	☐ MRI-based neuroimaging

Antibodies

Antibodies used

p44/42 MAPK (Erk1/2) [clone #L34F12] Mouse monoclonal antibody (CST #4696, lot #23) (1:1000), Phospho-p44/42 MAPK (Erk1/2) (Thr202/Tyr204) Rabbit antibody (CST #9101, lot #28) (1:1000), Akt (pan) [clone #40D4] Mouse monoclonal antibody (CST #2920, lot #8) (1:1000), Phospho-Akt (Ser473) Rabbit antibody (CST #9271, lot #14) (1:1000), GTPase HRAS [clone #Y132] Rabbit monoclonal antibody (Abcam #ab32417, lot #GR301687-2) (1:1000), KRAS [clone #3B10-2F2] Mouse monoclonal antibody (Sigma #WH0003845M1, lot #I7121-S2) (1:1000), NRAS Goat polyclonal antibody (Abcam #ab77392) (1:250), SOS1 Rabbit polyclonal antibody (CST #5890, lot #2) (1:1000), ARAF Rabbit polyclonal antibody (CST #4432, lot #2) (1:1000), BRAF [clone #F-7] Mouse monoclonal antibody (Santa Cruz #sc-5284, lot #L0919) (1:500), CRAF [clone #D5X6R] Mouse monoclonal antibody (CST #12552, lot #1) (1:500), Melanoma gp100 [clone #EP4863(2)] Rabbit monoclonal antibody (Abcam #ab137078) (1:100), IRDye 800CW Donkey anti-Goat IgG secondary antibody (LI-COR #926-32214, lot #C80717-07) (1:15000), IRDye 680RD Goat anti-Mouse IgG secondary antibody (LI-COR #926-68070, lot #C70613-15) (1:15000), IRDye 800CW Goat anti-Mouse IgG secondary antibody (LI-COR #926-32210, lot #C60405-05) (1:15000), IRDye 680RD Goat anti-Rabbit IgG secondary antibody (LI-COR #926-68071, lot #C80605-15) (1:15000), IRDye 800CW Goat anti-Rabbit IgG secondary antibody (LI-COR #926-32211, lot #C80426-08) (1:15000), Alexa Fluor 555 (Thermo Fisher #A-21428, lot #1858260) (4 µg/mL), Cre Recombinase (D7L7L) Rabbit monoclonal antibody (CST #15036, lot #2) (1:125), Ki67 Rabbit polyclonal antibody (Abcam #264429, lot #GR-275729-1) (1:1000), CD45 (D3F8Q) Rabbit monoclonal antibody (CST #70257S, lot #1) (1:200), Cleaved-Caspase-3 (D3E9) Rabbit monoclonal antibody (CST #9579S, lot #1) (1:250), IHC Select Secondary Goat Anti-Rabbit IgG antibody prediluted and biotinylated (Millipore #21537, lot #3604852), ImmPRESS-AP Horse anti-Rabbit IgG (ready-to-use) reagent (Vector Laboratories #MP-5401, lot #ZH0309)

Validation

The Cell signaling technologies (CST) antibody performance guarantee states that they validate all of their antibodies in-house in multiple research applications. We further confirmed the specificity of the ARAF, CRAF, and SOS1 antibodies by showing the loss of protein expression when cells were treated with shRNA specific for each target (see Figure 5). The phospho-p44/42 MAPK (ERK1/2), p44/42 MAPK (Erk1/2), Akt (pan), phospho-Akt (Ser473), SOS1, ARAF, and CRAF have been validated by CST to recognize both human and mouse homologs via immunoblot. Furthermore, the employed antibodies against Cre recombinase, CD45, and cleaved-Caspase-3 were validated by CST for the detection of murine proteins via immunohistochemical staining.

The Abcam antibodies used in this study are covered by Abcam's Abpromise guarantee, indicating that they have been tested and approved for the target species and application. Our results further validate the specificity of the NRAS and HRAS antibodies by demonstrating the loss of protein expression when each mRNA is depleted by shRNA (See Figure 5). Abcam antibodies used in this study have been validated for use in human and mouse samples. Specifically, the N-and HRAS antibodies are approved to recognize their respective human and mouse homologs via immunoblot. The gp100 and Ki67 antibodies are approved to recognize their murine homologs in murine tissues and tumors.

The Sigma KRAS antibody has been used for immunoblot analyses in other publications (PMID: 24351425). Our results further validate the specificity of this antibody by demonstrating the specific loss of KRAS protein detection upon the use of a KRAS-specific shRNA (see Figure 5). Sigma has validated that the KRAS antibody is effective in recognizing mouse KRAS by immunoblot.

The Santa Cruz BRAF antibody has been cited in over 200 publications. Our result further validated the specificity of this antibody by demonstrating loss of the protein following the treatment of cells with a BRAF-specific shRNA (See Figure 5). Santa Cruz has validated that the BRAF antibody is capable of recognizing mouse BRAF by immunoblot.

Eukaryotic cell lines

Policy information about cell lines

Cell line source(s)

HEK 293T cells (ATCC #CRL-3216), HEK 293AD cells (Agilent #240085), mouse embryonic fibroblasts (MEFs) and primary melanocytes isolated from TN mice were used in this study.

Authentication

STR analysis was performed on HEK 293T and HEK 293AD cell lines. Standard isolation techniques were used to generate MEFs from day E13.5 embryos and primary melanocytes from the skin of neonatal mice. However, formal authentication procedures were not performed on these cells because STR standards are not available. The genotype of all MEF and primary melanocyte cell lines was verified by PCR and/or immunoblot.

Mycoplasma contamination

HEK 293T and HEK 293AD cells routinely tested negative for mycoplasma contamination. The MEF and primary melanocyte cell lines were not tested for mycoplasma contamination.

Commonly misidentified lines (See ICLAC register)	HEK 293 cells are commonly misidentified as Hela cells so we performed short tandem repeat (STR) analysis to validate the authenticity of these cell lines.

Animals and other organisms

Policy information about studies involving animals; ARRIVE guidelines recommended for reporting animal research

Laboratory animals	Male and female C57BL/6 mice were used for this study. Animals were housed in temperature (72.5°F) and humidity (48.9%) controlled rooms with a 12-hour light cycle (lights on from 6 am-6 pm). 4-hydroxytamoxifen was topically administered to mice on post-natal days 1-2 followed by a single dose of UVB irradiation on post-natal day 3. Mice were monitored for tumor formation and tracked until reaching predetermined exclusion criteria approved by the Ohio State Institutional Care and Use Committee.
Wild animals	No wild animals were used in the study.
Field-collected samples	No field-collected samples were used in the study.
Ethics oversight	Animal work was performed in compliance with protocols approved by The Ohio State Institutional Care and Use Committee (Protocol #2012A00000134).

Note that full information on the approval of the study protocol must also be provided in the manuscript.

Flow Cytometry

Plots

Confirm that:

- [x] The axis labels state the marker and fluorochrome used (e.g. CD4-FITC).
- [x] The axis scales are clearly visible. Include numbers along axes only for bottom left plot of group (a 'group' is an analysis of identical markers).
- [x] All plots are contour plots with outliers or pseudocolor plots.
- [x] A numerical value for number of cells or percentage (with statistics) is provided.

Methodology

Sample preparation	Passage three TN61X/X MEFs were infected with Ad5-CMV-Cre-eGFP to induce NRAS expression as described for our in vitro studies. The cells were then cultured for five days. MEFs were placed in DMEM containing 1% penicillin-streptomycin and 1% glutamine for five hours prior to adding 0.01 mM 5-ethynyl-2-deoxyuridine (EdU) to the media. MEFs were labeled with EdU for an additional five hours and then harvested and fixed with 4% paraformaldehyde. Fixed cells were permeabilized with saponin in 1% BSA 1x PBS. Click-iT chemistry was used to label the incorporated EdU with Chromeo 642. Here, the cells were incubated for 30 minutes in Click-iT reaction cocktail containing 2 mM CuSO4, 50 mM ascorbic acid and 50 nM Chromeo 642 azide dye (Active Motif 15288) diluted in 1x PBS. Cells were washed in PBS before analysis.
Instrument	A BD LSR Fortessa flow cytometer was used for this analysis.
Software	Analysis of EdU positivity was performed using FlowJo software version 10.
Cell population abundance	MEF cell lines were isolated from embryos on embryonic day E13.5 using standard isolation techniques. These cells were cultured as monolayers in vitro until EdU labeling for flow cytometry.
Gating strategy	The initial population of MEFs was selected by gating based on forward (FSC-A) and side (SSC-A) scatter. Next, cell doublets were removed by gating for single cells in a FSC-H by FSC-A plot. Finally, a histogram of counts by APC-A intensity was used to determine the percent of EdU positive cells in each population of MEFs. The EdU negative gate was set using a no EdU control sample.

- [x] Tick this box to confirm that a figure exemplifying the gating strategy is provided in the Supplementary Information.
